# Cancer Manipulates Adjacent Adipose Tissue to Exploit Fatty Acids via HIF‐1α/CCL2/PPARα Axis: A Metabolic Circuit to Support Tumor Progression

**DOI:** 10.1002/advs.202515186

**Published:** 2025-10-29

**Authors:** Jeong‐Eun Yun, Jieun Seo, Jiwon Koh, Seock‐Ah Im, Ki Yong Hong, Yeseon Son, Do‐Won Jeong, Junji Fukuda, Jong‐Wan Park, Yang‐Sook Chun

**Affiliations:** ^1^ Department of Biomedical Sciences Seoul National University College of Medicine Seoul 03080 Republic of Korea; ^2^ Department of Physiology Seoul National University College of Medicine Seoul 03080 Republic of Korea; ^3^ Faculty of Engineering Yokohama National University Yokohama 240‐8501 Japan; ^4^ Department of Pathology Seoul National University Hospital Seoul 03080 Republic of Korea; ^5^ Cancer Research Institute Seoul National University Seoul 03080 Republic of Korea; ^6^ Seoul National University Hospital Cancer Research Institute Seoul National University College of Medicine Seoul National University Seoul 03080 Republic of Korea; ^7^ Department of Plastic and Reconstructive Surgery Seoul National University Hospital Seoul 03080 Republic of Korea; ^8^ Department of Cell Biology Harvard Medical School Boston MA 02115 USA; ^9^ Ischemic/Hypoxic Disease Institute Seoul National University College of Medicine Seoul 03080 Republic of Korea

**Keywords:** cancer, CCL2, HIF‐1α, obesity, PPARα

## Abstract

Rising obesity rates are closely linked to higher risk of cancer, yet the underlying mechanisms are not fully understood. It is previously reported that fatty acids (FAs) released from cancer‐associated adipose tissue enhance hypoxia‐inducible factor‐1α (HIF‐1α) expression in cancer cells, promoting tumor progression. Here, it is elucidated that cancer cells manipulate adjacent adipose tissue by secreting C‐C chemokine ligand2 (CCL2) to exploit FAs. Activation of HIF‐1α induced by FA influx increases CCL2 expression in cancer cells, which subsequently leads to lipolysis in nearby adipose tissue by activating peroxisome proliferator‐activated receptor alpha (PPARα) signaling. This activation in adipose tissue results in the release of FAs into the tumor microenvironment. The increased lipid supply to tumor reactivates the FA/HIF‐1α/CCL2 axis in cancer cells, further accelerating tumor growth and CCL2 secretion. This establishes a positive feedback loop between tumor and adjacent adipose tissue, which enhances cancer progression. This crosstalk is validated by using a polydimethylsiloxane‐based 3D coculture system and in vivo models. In obese mice, this reciprocal signaling accelerated tumor progression, whereas intra‐tumoral injection of CCL2‐neutralizing antibody significantly suppressed it. These findings reveal a metabolic circuit for tumor survival and disrupting this interaction may provide promising therapeutic targets, particularly for obese cancer patients.

## Graphical abstract

Cancer cells drive adjacent adipose tissue to release fatty acids by secreting CCL2, which activates PPARα‐dependent lipolysis. The resulting fatty acid influx amplifies HIF‐1α/CCL2 signaling, establishing a positive feedback loop that fuels tumor growth. This cancer–adipose crosstalk, validated in 3D coculture, obese mouse models, and breast cancer tissues, underscores CCL2 blockade as a potential therapy for obesity‐associated cancers.

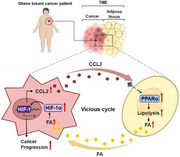

## 1 Introduction

The growing rate of obesity has become a global health concern, as excessive weight gain is associated with an increased risk of various diseases, including cancer.^[^ ^1^, ^2^ ^]^ Obesity is a well‐established risk factor for multiple cancer types, including breast, prostate, pancreatic, and colorectal cancers, and is associated with increased mortality.^[^ ^2^, ^3^, ^4^ ^]^ In particular, obesity has been recognized as a major preventable cause of breast cancer since Abe et al. first reported its critical impact on breast cancer diagnosis in 1976.^[^ ^5^ ^]^ Moreover, recent meta‐analyses have shown that obese women diagnosed with breast cancer have approximately a 30% higher risk of recurrence or mortality compared to women of normal weight.^[^ ^6^, ^7^ ^]^ These findings have prompted intensive research efforts to better understand the influence of obesity on breast cancer progression.^[^ ^8^, ^9^ ^]^ Nevertheless, over the past decade, research into obesity and cancer has predominantly centered on its immunological aspects, particularly the suppression of anti‐tumor immunity by obese condition.^[^ ^10^, ^11^ ^]^ Thus, the underlying mechanisms directly linking obesity to cancer remain complex and not yet fully understood. In this study, we elucidate the impact of obesity on cancer by presenting a reciprocal signaling between tumor and adipose tissue, focusing on a previously underexplored aspect.

Unlike normal cells, cancer cells are characterized by rapid and continuous proliferation. This uncontrolled cell division requires a constant supply of essential cellular components to sustain growth and ensure survival. Consequently, cancer cells exhibit a heightened demand for fatty acids (FAs), which serve as essential components for membrane synthesis and as crucial energy sources to support rapid proliferation and elevated metabolic activity.^[^ ^12^ ^]^ Thus, to fuel this continuous growth, cancer cells undergo metabolic reprogramming, utilizing FAs from tumor microenvironment (TME). Among the various components of the TME, we focused on adipose tissue directly adjacent to the tumor, highlighting the possibility that tumors exploit adipose tissue as an energy reservoir. In this context, we aimed to elucidate the mechanism by which tumors colonize adipose tissue and induce lipolysis to obtain FAs.

Hypoxia‐inducible factor‐1 alpha (HIF‐1α) is an essential transcription factor that supports cancer cell survival by up‐regulating the expression of genes involved in proliferation and migration under hypoxia.^[^ ^13^, ^14^ ^]^ In our previous studies, it was confirmed that FAs increase HIF‐1α activity in liver, colon, prostate, and breast cancers.^[^ ^15^, ^16^ ^]^ Meanwhile, HIF‐1α enhances FAs uptake by inducing the expression of Fatty Acid Binding Protein3 (FABP3), FABP7, and Perilipin‐2 in breast cancer cells, which are critical for cell growth and survival. Collectively, HIF‐1α‐mediated FA uptake leads to FA accumulation, which in turn amplifies HIF‐1α activity, exacerbating cancer. Nevertheless, in this process, the lipid accumulation is due to FAs uptake, but not de novo synthesis.^[^ ^17^ ^]^ Therefore, uncovering how cancer cells continuously acquire FAs from TME has become a key focus of our research.

Here, we investigated how cancer cells secure a continuous supply of FAs from TME to sustain their progression. Focusing on tumor‐adjacent adipose tissue as a potential FA reservoir, we employed a 3D coculture system along with in vivo models. Consequently, our findings demonstrated that cancer cells secrete the chemokine C‐C motif ligand 2 (CCL2) as a signaling molecule, which induces lipolysis in neighboring adipose tissue, thereby promoting FA release and tumor growth. Building upon our previous study, we further revealed that this reciprocal crosstalk establishes a positive feedback loop between the two tissues, offering a mechanistic explanation for how tumor progression is exacerbated under obese conditions.

## 2 Results

### 2.1 Tumor‐Adjacent Adipose Tissue Is Reduced by the Tumor and Further Depleted under a High‐Fat Diet, Facilitating Tumor Progression

**Figure 1:**
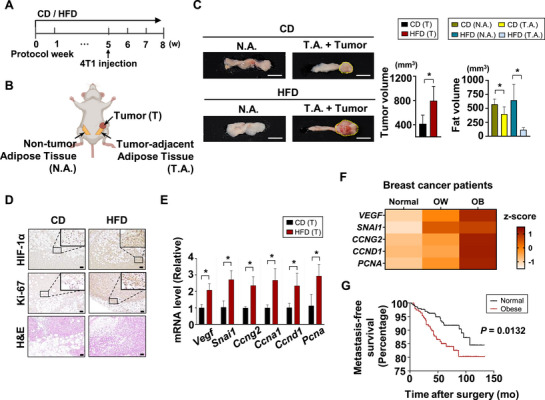
Tumor‐Adjacent Adipose Tissue Is Reduced by the Tumor and Further Depleted under a High‐Fat Diet, Facilitating Tumor Progression. A) Schematic diagram of the in vivo model. Starting from 4 weeks of age, all the mice were fed either a chow diet (CD) or high‐fat diet (HFD). At week 5 of feeding, 4T1 cells were orthotopically injected into the fourth mammary fat pad of female NOD/SCID mice. Mice were euthanized three weeks following cancer cell transplantation. (n=5 independent animals in each group) B) Schematic illustration showing the tumor, tumor‐adjacent adipose tissue (T.A.), and contralateral non‐tumor adipose tissue (N.A.) collected for subsequent morphological and molecular analyses. C) Representative images of N.A. and T.A. with tumor obtained from CD and HFD groups. Scale bar:10 mm. Tumor and fat volumes (mm^3^) were calculated using the formula: (width × length^2^)/2. Mean ± SD (n =5 in each group); ^*^ *p* < 0.05. D) Hematoxylin and Eosin (H&E) staining and immunohistochemical analysis (IHC) of tumor sections using the indicated antibodies and DAB staining. Scale bar = 100 µm. (n=5 in each group) E) qRT‐PCR analysis of tumor tissues revealed the expression of proliferation‐associated genes, including HIF‐1α downstream targets. All qRT‐PCR data were normalized to 18S RNA. Mean ± SD (n=5 in each group) ^*^ *p* < 0.05. F) In an analysis of 572 breast cancer patients from the GEO dataset GSE93601, expression of proliferation‐related genes was examined in breast tumor tissues stratified by body mass index (BMI) into normal, overweight (OW), and obese (OB) groups. BMI groups were classified as follows: Normal (20–24.9 kg m^−2^, n=265), OW (25–29.9 kg m^−2^, n=76), OB (≥30 kg m^−2^, n=131). G) Kaplan‐Meier curve of metastasis‐free survival of normal or obese breast cancer patients. BMI groups were classified as follows: Normal (18.5–24.9 kg m^−2^, n=193) OB (≥25 kg m^−2^, n=297) Log rank P=0.0132. The P‐value was calculated using the log‐rank test.

Obesity has been reported to worsen breast cancer.^[^ ^5^ ^]^ To study its impact on cancer progression, we divided mice into a chow diet (CD) group and a high‐fat diet (HFD) group. After 5 weeks, when a significant weight difference was noted (Figure S1A, Supporting Information), we injected 4T1 breast cancer cells into the fourth mammary fat pad (**Figure** **1**A). Three weeks after the injection, we harvested the tumors along with the surrounding adipose tissues, referred to as tumor‐adjacent adipose tissue (T.A.). Adipose tissues from the contralateral side, which contained no cancer cells, were also collected and designated as non‐tumor adipose tissue (N.A.), as shown in Figure 1B. The results clearly indicate that tumors in the mice fed a HFD were larger than those in the group consuming a standard CD. This confirms a significant increase in tumor growth associated with the HFD. Interestingly, the size of the T.A. was smaller compared to that of N.A. on the opposite side. This suggests that the tumor may utilize lipids stored in the nearby adipose tissue to promote its own growth, thereby decreasing the volume of the surrounding adipose tissue. Furthermore, the reduction in the volume of T.A. compared to N.A. was markedly greater in the HFD group than in the CD group (Figure 1C; Figure S1B, Supporting Information). These findings indicate that the tumor's exploitation of lipids may be more pronounced under conditions of high lipid availability, such as obesity induced by a HFD. Immunohistochemistry (IHC) results demonstrated a significant increase in HIF‐1α expression in tumors from mice that were fed an HFD. This increase was associated with elevated levels of Ki‐67, a well‐known marker of tumor proliferation (Figure 1D). Moreover, mRNA expression levels of proliferation‐associated genes, including HIF‐1α target genes, were higher in HFD‐fed mice compared to CD‐fed mice, indicating accelerated tumor growth in the HFD group (Figure 1E). Supporting the in vivo data, analysis of 572 breast cancer patients from a GEO data base (GSE93601), categorized by body mass index (BMI) into normal, overweight (OW), and obese (OB) groups, revealed that the expression levels of these proliferation‐related genes in breast tumor tissues progressively increased with higher BMI (Figure 1F). Consistent with the in vivo findings, obesity in breast cancer patients was associated with reduced metastasis‐free survival (Figure 1G). In earlier studies, we demonstrated that increased lipid availability promotes tumor progression through upregulation of HIF‐1α.^[^ ^15^, ^16^ ^]^ Our current findings confirm that obesity induced by a HFD leads to an increase in HIF‐1α expression level in tumors, further accelerating tumor progression as we have shown in previous research. Simultaneously, our results indicate that the adipose tissue adjacent to the tumor was reduced in size, with this reduction becoming more pronounced under HFD conditions, which also promoted tumor growth (Figure 1C,D). Thus, tumor cells may upregulate HIF‐1α expression by utilizing FAs extorted from nearby adipose tissues, thereby reducing the adjacent adipose tissue volume and promoting cancer progression. In addition, a more distinct size difference between N.A. and T.A. observed under HFD conditions suggests that increased systemic lipid availability further facilitates the tumor's exploitation of FAs from neighboring adipose tissues (Figure 1C).

### 2.2 FAs from Adipose Tissues Drive HIF‐1α‐Mediated CCL2 Secretion in Cancer Cells

**Figure 2:**
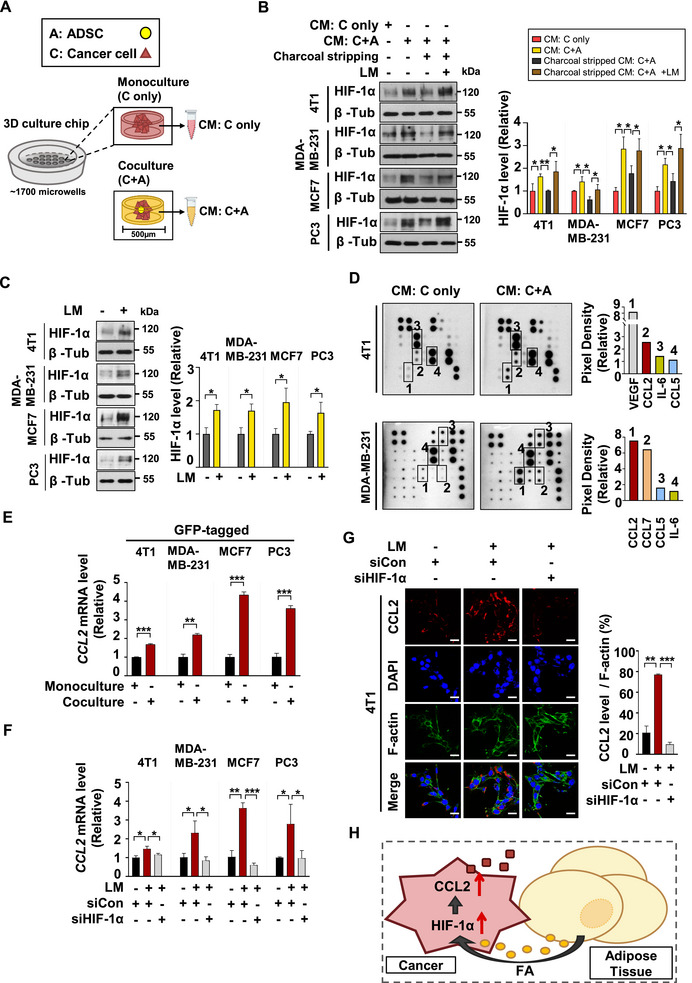
FAs from Adipose Tissues Drive HIF‐1α‐Mediated CCL2 Secretion in Cancer Cells. A) Structural and schematic illustration of PDMS‐3D culture chips. Total 5 × 10^5^cells were seeded onto the chips. For monoculture, only cancer cells were seeded, whereas for coculture, cancer cells and adipocyte‐derived stem cells (ADSCs) were seeded at a 10:1 ratio. Conditioned media (CM) were collected after 48 h from monocultured 3D culture chips (CM: C only) and cocultured 3D culture chips (CM: C+A). B) HIF‐1α protein levels were analyzed by western blotting following 24 h of treatment with each CM. To deplete lipids, the coculture‐derived CM were incubated with activated charcoal for 8 h following collection, as indicated. To restore lipids, 2% lipid mixture (LM) was added to the charcoal stripped coculture‐derived CM. Mean ± SD (n = 3); ^*^, *p* < 0.05. C) Western blotting was performed to assess HIF‐1α protein levels after 24‐h treatment with 2% LM to cancer cells. Mean ± SD (n = 3); ^*^, *p* < 0.05. D) A cytokine array was performed with the CM from monocultured cancer cells (CM: C only) and the CM from cancer cells cocultured with ADSCs (CM: C+A). The levels of cytokines regulated by HIF‐1α were compared between monoculture‐derived CM (CM: C only) and coculture‐derived CM (CM: C+A), and the relative increases in coculture‐derived CM (CM: C+A) were visualized in a graph. The mouse breast cancer cells (4T1) were cocultured with mouse ADSCs and human breast cancer cells (MDA‐MB‐231) were cocultured with human ADSCs. E) GFP‐tagged stable cancer cell lines were monocultured or cocultured with ADSCs. The mouse cancer cell line (4T1) was cocultured with mouse ADSCs and human cancer cell lines (MDA‐MB‐231, MCF7, and PC3) were cocultured with human ADSCs. The cocultured cancer cells were sorted as indicated in Figure S2B (Supporting Information). The CCL2 mRNA levels between cocultured cancer cells and monocultured cancer cells were compared by qRT‐PCR. Mean ± SD (n = 3); ^*^, *p* < 0.05; ^**^, *p* < 0.001; ^***^, *p* < 0.0001. F) Cancer cells were transfected with si‐Con or si‐HIF‐1α and treated with PBS or 2% LM for 48 h. The CCL2 mRNA levels were measured by qRT‐PCR. Mean ± SD (n = 3); ^*^, *p* < 0.05; ^**^, *p* < 0.001; ^***^, *p* < 0.0001. G) Immunofluorescence (IF) analysis was performed using the indicated antibodies following transfection with si‐Con or si‐HIF‐1α and treatment with PBS or 2% LM for 48 h. Nuclei were stained with DAPI, and F‐actin was stained with Alexa Fluor 488–phalloidin. Scale bar = 20 µm. Mean ± SD (n = 3); ^**^, *p* < 0.001; ^***^, *p* < 0.0001. H) Diagram of the FA/HIF‐1α/CCL2 axis in cancer.

Based on the results shown in Figure 1, we speculated that under increased FA supply, cancer cells accumulating HIF‐1α might actively secrete a signaling molecule to stimulate lipolysis in nearby adipose tissue. To identify this messenger from cancer cells, we employed a 3D coculture system that mimics in vivo conditions. In our previous study, we clearly established the use of a custom‐designed 3D coculture chip made of polydimethylsiloxane (PDMS), which effectively supplies oxygen from the bottom of the chips (**Figure** **2**A).^[^ ^15^, ^16^ ^]^ Building on our in vivo findings, we rigorously investigated the impact of lipids derived from adipose tissues on cancer cells by treating them with conditioned media (CM) obtained from either monocultured cancer cells or cancer cells cocultured with adipocyte‐derived stem cells (ADSCs). After treatment of the CM, HIF‐1α expression levels in the cancer cells were assessed. Accordingly, treatment with CM derived from cocultured cancer cells led to increased HIF‐1α levels compared to monoculture‐derived CM. However, this increase was eliminated when the lipids in coculture‐derived CM was removed by charcoal. Restoring HIF‐1α expression was achieved by adding a lipid mixture (LM) to the charcoal‐stripped coculture‐derived CM. These results indicate that adipose tissue‐derived lipids are essential for upregulating HIF‐1α in cancer cells (Figure 2B). Furthermore, treatment with LM to cancer cells for 24 h led to upregulation of HIF‐1α and genes associated with cell proliferation and survival, suggesting an acceleration of cancer progression (Figure 2C; Figure S2A, Supporting Information). Therefore, we asserted that the FA‐induced HIF‐1α induction strongly promotes the secretion of specific cytokines from cancer cells, functioning as crucial signaling molecules. Our cytokine array analysis reveals that among the HIF‐1α‐regulated cytokines (CCL2, CCL5, CCL7, IL‐6, and VEGF),^[^ ^18^, ^19^, ^20^, ^21^ ^]^ CCL2 secretion was significantly elevated in the CM from the mouse breast cancer cell line (4T1) cocultured with mouse ADSCs and the human breast cancer cell line (MDA‐MB‐231) cocultured with human ADSCs, compared to their respective monoculture‐derived CM controls (Figure 2D). To determine whether the increased CCL2 in coculture‐derived CM was secreted by cancer cells, we tagged the cancer cells with GFP and cocultured them with ADSCs in the 3D coculture system. The GFP‐tagged cancer cells were then isolated using fluorescence‐activated cell sorting (FACS), as shown in Figure S2B (Supporting Information). We compared CCL2 mRNA levels in the sorted cancer cells grown in coculture with ADSCs versus those in monoculture. Consequently, CCL2 expression was significantly higher in the cocultured cancer cells. This indicates that the presence of lipids from ADSCs boosts CCL2 expression and its secretion from cancer cells into the coculture‐derived CM (Figure 2E). To confirm the FAs/HIF‐1α/CCL2 axis, 2% LM was added to cancer cells transfected with either si‐Con or si‐HIF‐1α (Figure S2C, Supporting Information), and then CCL2 mRNA levels were measured. The result shows that the LM‐induced upregulation of HIF‐1α led to an increase in CCL2 expression. However, this effect was not observed in cancer cells where HIF‐1α was knocked down, indicating that CCL2 is a downstream of HIF‐1α (Figure 2F). This result was confirmed by immunofluorescence (IF) staining for CCL2 expression (Figure 2G). These findings indicate that adipose tissue‐derived FAs elevate HIF‐1α levels in tumors, leading to a significant upregulation of CCL2 expression and its secretion from the cancer cells. Thus, our data support the existence of a functional FAs/HIF‐1α/CCL2 axis in cancer cells under increased FA supply (Figure 2H).

### 2.3 CCL2‐Activated PPARα Signaling Drives Lipolysis and FA Release

**Figure 3:**
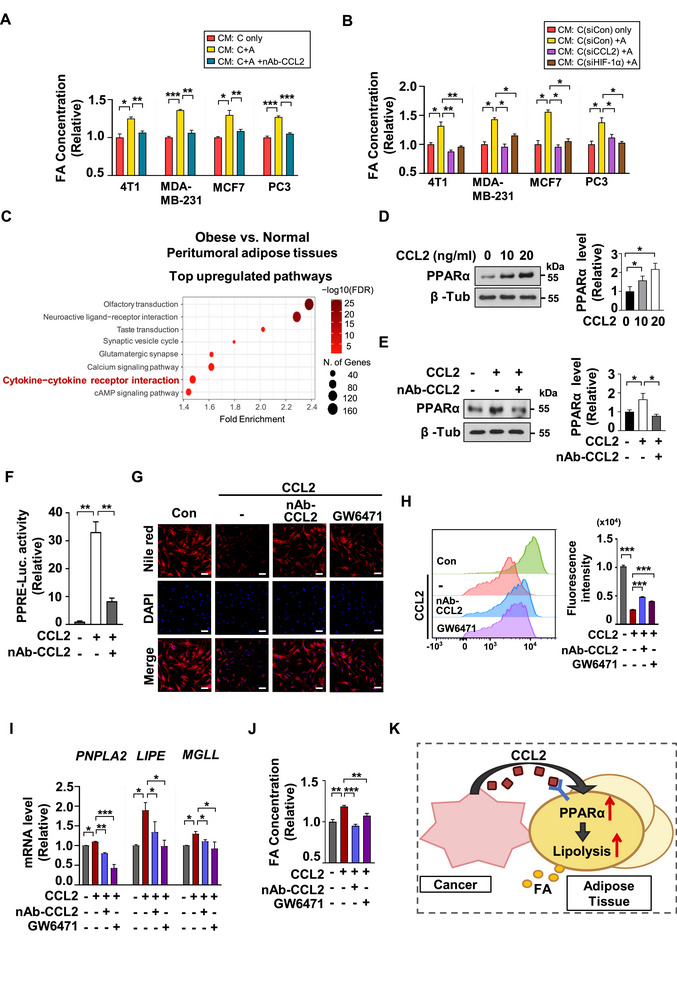
CCL2‐Induced PPARα Accumulation Drives Lipolysis and FA Release. A,B) Using the indicated FA quantification kit, we measured the FA concentrations in CM collected from the experiment shown in Figure S3,E (Supporting Information). Mean ± SD (n = 3); ^*^, *p* < 0.05; ^**^, *p* < 0.001; ^***^, *p* < 0.0001. C) In the GEO dataset comprising tumor‐adjacent adipose tissues from 34 breast cancer patients (GSE153316), gene expression profiles were compared between normal‐weight and obese groups. The groups were classified as follows: Normal (20–24.9 kg m^−2^, n=21), Obese (≥25 kg m^−2^, n=13). D) ADSCs were treated with CCL2 for 2 h with the indicated concentrations and the protein levels were analyzed by western blotting with indicated antibodies. Mean ± SD (n = 3); ^*^, *p* < 0.05. E) ADSCs were treated with CCL2 (20 ng mL^−1^) and nAb‐CCL2 (1 µg mL^−1^) for 2 h. Proteins were analyzed by western blotting with indicated antibodies. Mean ± SD (n = 3); ^*^, *p* < 0.05. F) ADSCs were transfected with PPRE‐Luc plasmid and treated with CCL2 (20 ng mL^−1^) and nAb‐CCL2 (1 µg mL^−1^) for 24 h. Mean ± SD (n = 3); ^*^, *p* < 0.05; ^**^, *p* < 0.001. G) Lipid accumulation was estimated by Nile red staining. ADSCs were treated with CCL2 (20 ng mL^−1^), nAb‐CCL2 (1 µg mL^−1^), or GW6471 (PPARα inhibitor, 1 µm) for 24 h. After staining with Nile Red and DAPI, the cells were visualized by fluorescence microscopy. Scale bar = 100 µm H) FACS analysis of Nile Red stained cells. The graph (right) indicate the mean fluorescence intensity. Mean ± SD (n = 3) ^***^, *p* < 0.0001. I) ADSCs were treated with CCL2 (20 ng mL^−1^), nAb‐CCL2 (1 µg mL^−1^), or GW6471 (1 µm) for 24 h as indicated. The mRNA levels were quantified by qRT‐PCR. Mean ± SD (n = 3); ^*^, *p* < 0.05; ^**^, *p* < 0.001; ^***^, *p* < 0.0001. J) FA concentrations in CM collected after 24 h of treatment with CCL2 (20 ng mL^−1^), nAb‐CCL2 (1 µg mL^−1^), or GW6471 (1 µm) to ADSCs were measured using the indicated FA quantification kit. Mean ± SD (n = 3); ^*^, *p* < 0.05; ^**^, *p* < 0.001; ^***^, *p* < 0.0001. K) Diagram of the CCL2/PPARα/FA axis in adipose lipolysis.

To assess whether cancer cell‐derived CCL2 regulates lipid metabolism in adipose tissue, we used a neutralizing antibody (nAb‐CCL2) to inhibit CCL2 during 3D coculture of cancer cells and ADSCs. The CM were subsequently collected from the 3D culture chips shown in Figure S3A (Supporting Information). The concentration of fatty acids (FAs) in the coculture‐derived CM was significantly higher than those in the monoculture‐derived CM. However, this elevated FA concentration was effectively reduced to baseline levels upon CCL2 inhibition with nAb‐CCL2 (**Figure** **3**A). In addition, we cocultured cancer cells with ADSCs after silencing CCL2 or HIF‐1α in the cancer cells (Figure S3C, Supporting Information), thereby inhibiting CCL2 secretion (see Figure S3D,E, Supporting Information). Following this process, CM from the chips shown in Figure S3E (Supporting Information) were collected. The elevated FA concentration in the CM from the coculture of control cancer cells and ADSCs decreased when the cancer cells were transfected with siCCL2 or siHIF‐1α before coculture (Figure 3B). These findings suggest that suppressing cancer cell‐derived CCL2 reduced FA release from ADSCs, indicating that CCL2 secretion from tumors plays a key role in inducing FA release from adjacent adipose tissues. To examine the impact of cancer cell‐derived CCL2 on adipose tissue, we conducted gene ontology analysis. A GEO dataset of peritumoral adipose tissues from breast cancer patients (GSE153316) was divided into normal‐weight and obese groups based on BMI. Comparison of gene expression profiles revealed that genes related to the cytokine–cytokine receptor interaction pathway were upregulated in the adipose tissues of obese patients compared to normal‐weight patients (Figure 3C). Among the upregulated genes in the peritumoral adipose tissues of obese breast cancer patients, C‐C chemokine receptor 2 (CCR2), a receptor for CCL2, was identified (Figure S3H, Supporting Information). Our earlier findings indicate that increased FA supply activates the FA/HIF‐1α/CCL2 axis in cancer cells. The upregulation of CCR2 in peritumoral adipose tissue indicates potential crosstalk through the CCL2–CCR2 signaling pathway. Based on the altered FA concentrations in the CM, as shown in Figure 3A,B, we explored how CCL2 from cancer cells affects lipid metabolism in adipose tissue. We analyzed the same GEO dataset to identify transcription factors related to lipid metabolism that are influenced by CCL2‐CCR2 signaling. Our analysis aimed to determine which signaling pathways of these transcription factors showed a positive correlation with CCR2 expression in the adipose tissue of obese patients. Our findings revealed that, among the key lipid metabolism‐related transcription factors, only the downstream pathways of the proliferator‐activated receptor (PPAR) family showed a significant positive correlation with a valid FDR (<0.25) (Figure S3I, Supporting Information). Consequently, we treated ADSCs with CCL2 and monitored the expression of PPAR members (Figure S3J, Supporting Information). Among them, PPARα showed a marked and early increase, leading us to focus on PPARα as a target that is upregulated by CCL2‐CCR2 signaling. Additionally, the expression of PPARα increased in a dose‐dependent manner in response to CCL2 treatment (Figure 3D). The upregulation was unequivocally reversed when CCL2 was inhibited by nAb‐CCL2 treatment or when CCR2 was knocked down in ADSCs (Figure 3E; Figure S3K, Supporting Information). This clearly confirms that CCL2 drives the accumulation of PPARα in ADSCs. As PPARα binds to the peroxisome proliferator response element (PPRE) in the promoter region of its downstream target genes,^[^ ^22^ ^]^ we transfected ADSCs with PPRE‐Luc plasmid to assess the transcriptional activity of PPARα with luciferase assay.^[^ ^23^ ^]^ The transfected ADSCs were subsequently treated with CCL2 and nAb‐CCL2 as indicated. CCL2 treatment significantly increased PPRE‐luciferase activity, while nAb‐CCL2 reduced it, confirming that CCL2 enhances PPARα activity (Figure 3F). To evaluate the effects on lipid metabolism, ADSCs were treated with CCL2, followed by nAb‐CCL2 or a PPARα inhibitor GW6471. Subsequently, intracellular lipid accumulation was evaluated using Nile Red staining. The result shows that CCL2 treatment promoted lipolysis in ADSCs, leading to reduced lipid accumulation. However, this effect was reversed by CCL2 neutralization or PPARα inhibition, restoring intracellular lipid content (Figure 3G). Flow cytometry analysis further confirmed these changes in lipid accumulation through Nile Red fluorescence measurement (Figure 3H). The qPCR analysis reveals that CCL2 treatment significantly boosts the expression of key lipolysis‐related genes, *PNPLA2, LIPE, and MGLL*. Conversely, their expression is suppressed by nAb‐CCL2 or GW6471 (Figure 3I). Additionally, silencing CCR2 or PPARα with siRNAs effectively disrupts the CCL2/CCR2/PPARα pathway, resulting in reduced expression of these genes induced by CCL2 treatment (Figure S3L, Supporting Information). This evidence emphasizes the critical role of the CCL2/CCR2/PPARα axis in lipolysis regulation. Measurement of FA levels in the CM from the experiments shown in Figure 3I clearly indicates that CCL2‐induced lipolysis enhanced FA release, a response that was effectively reduced by treatment with nAb‐CCL2 or GW6471 (Figure 3J). Similarly, FA levels in CM from the experiments presented in Figure S3L (Supporting Information) shows that CCL2‐induced increase in FA release was attenuated when either CCR2 or PPARα was knocked down (Figure S3M, Supporting Information). These results clearly demonstrate that the CCL2/CCR2/PPARα axis in adipose tissue plays a crucial role in enhancing lipolysis and promoting FA release (Figure 3K).

### 2.4 CCL2 Increases PPARα Stability via p‐ERK Signaling Pathway

**Figure 4:**
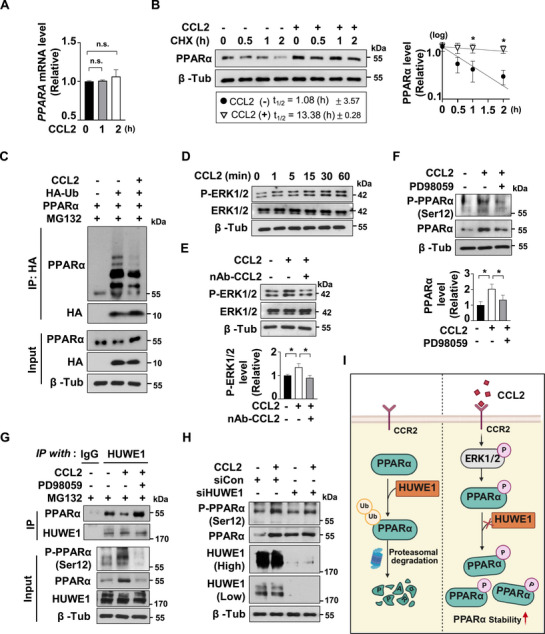
CCL2 Increases PPARα Stability via p‐ERK Pathway. A) The PPARα mRNA level was quantified by qRT‐PCR following treatment of human ADSC with CCL2 (20 ng mL^−1^) for the indicated time points. Mean ± SD (n = 3); n.s., not significant. B) ADSCs were pre‐treated with or without CCL2 for 4 h and incubated with cycloheximide (CHX) (100 µm) for the indicated time points. Cell lysates were subjected to western blot using the indicated antibodies (left). Band intensities on the blots were analyzed using ImageJ and plotted (right). Mean ± SD (n = 3); ^*^, *p* < 0.05. C) HA‐Ub and PPARα plasmid were transfected into ADSCs, and CCL2 (20 ng mL^−1^) was treated for 4 h and incubated with MG132 (10 µm) for 2 h. Cell lysates were subjected to immunoprecipitation (IP) using HA‐affinity beads, and the precipitated proteins were analyzed by western blotting. D) CCL2 (20 ng mL^−1^) was treated to ADSCs for the indicated time points. The protein levels were analyzed by western blotting with indicated antibodies. E) CCL2 (20 ng mL^−1^) and nAb‐CCL2 (1 µg mL^−1^) were treated for 30 min and the protein levels were analyzed by western blotting with indicated antibodies. Mean ± SD (n = 3); ^*^, *p* < 0.05. F) ADSCs were pre‐treated with PD98059 (20 µm) for 4 h, followed by CCL2 (20 ng mL^−1^) treatment for 2 h. Western blotting was performed on cell lysates using the indicated antibodies. Mean ± SD (n = 3); ^*^, *p* < 0.05. G) ADSCs were pre‐treated with PD98059 (20 µm) for 4 h, followed by treatment with CCL2 (20 ng mL^−1^) for 4 h and MG132 (10 µM) for 2 h. Cell lysates were subjected to IP using a HUWE1 antibody, and the precipitated proteins were analyzed by western blotting with indicated antibodies. H) ADSCs were transfected with siCon or siHUWE1 and subsequently treated with CCL2 (20 ng mL^−1^) for 2 h. The protein levels were analyzed by western blotting with indicated antibodies. I) Schematic illustration of PPARα regulation via the CCL2/p‐ERK/p‐PPARα axis.

Figure 3D,E shows that CCL2 treatment leads to an increase in PPARα protein levels. To explore the mechanism behind this increase, we measured PPARα mRNA levels by conducting qPCR in CCL2‐treated ADSCs, but as **Figure** **4**A indicates, there were no significant changes in mRNA expression. When ADSCs were pre‐treated with or without CCL2 and subsequently incubated with cycloheximide (CHX) for the indicated time points, CCL2 treatment was found to increase the half‐life of PPARα protein, indicating improved protein stability (Figure 4B). To confirm that CCL2 regulates PPARα ubiquitination, we transfected ADSCs with HA‐tagged ubiquitin and conducted immunoprecipitation with an anti‐HA antibody. CCL2 treatment significantly reduced PPARα ubiquitination, demonstrating that CCL2 effectively inhibits the ubiquitination‐dependent proteasomal degradation of PPARα (Figure 4C). This finding highlights the crucial role of CCL2 in regulating PPARα stability. To identify signaling pathways activated by the CCL2–CCR2 axis, we examined pathways previously reported to be associated with CCL2–CCR2 signaling (Figure S4A, Supporting Information).^[^ ^24^, ^25^ ^]^ We observed an increase in phosphorylated ERK (p‐ERK) sooner than the upregulation of PPARα after CCL2 treatment (Figure 4D). This activation was further confirmed by its reduction after nAb‐CCL2 treatment or siCCR2 transfection, thereby supporting the role of the ERK pathway in mediating CCL2 effects (Figure 4E; Figure S4B, Supporting Information). Given that PPARα phosphorylation enhances its stability by reducing ubiquitination, we investigated whether its phosphorylation is mediated by p‐ERK signaling in response to CCL2.^[^ ^26^ ^]^ ADSCs were pre‐treated with PD98059, an ERK inhibitor that suppresses p‐ERK activation, before CCL2 treatment (Figure S4C, Supporting Information). CCL2 treatment increased phosphorylated PPARα (p‐PPARα) at Ser12 and total PPARα protein levels. However, this phosphorylation was reduced when pre‐treated with the ERK inhibitor (PD98059), suggesting that CCL2 promotes Ser12 phosphorylation of PPARα through the activation of the p‐ERK signaling pathway (Figure 4F). Figure S4D (Supporting Information) shows that CCL2 reduces PPARα ubiquitination, a process that is reversed by inhibiting p‐ERK signaling, suggesting that the CCL2/p‐ERK/p‐PPARα pathway enhances PPARα stability. We also examined how PPARα phosphorylation affects its interaction with HUWE1, an E3 ligase.^[^ ^27^ ^]^ Immunoprecipitation with a HUWE1 antibody revealed that CCL2 treatment decreased the HUWE1‐PPARα interaction, which was restored by inhibiting p‐ERK signaling with pre‐treatment of PD98059. These findings indicate that phosphorylation of PPARα through the CCL2/p‐ERK pathway reduces its binding to HUWE1, thereby reducing proteasomal degradation and promoting PPARα accumulation (Figure 4G). Furthermore, the knockdown of HUWE1 increased total PPARα levels regardless of CCL2 treatment, while an increase in p‐PPARα was observed only with CCL2 treatment (Figure 4H). This supports the conclusion that CCL2 enhances PPARα stability by diminishing its interaction with HUWE1, resulting in increased accumulation (Figure 4I).

### 2.5 Blocking CCL2/CCR2/PPARα Axis Reduces Adipose‐lipolysis and FA Release, Suppressing Cancer Progression

**Figure 5:**
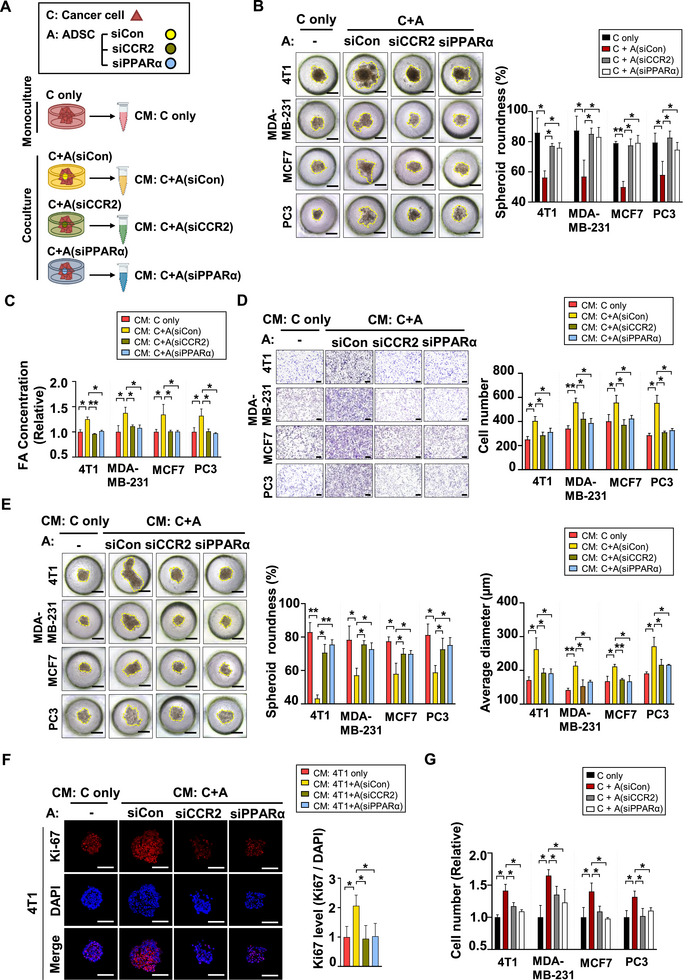
Blocking CCL2/CCR2/PPARα Axis Reduces Adipose‐lipolysis and FA Release, Suppressing Cancer Progression. A) Schematic diagram for Figure 5B of mono‐ or co‐culture of cancer cells with transfected ADSCs in 3D culture system. B) Single‐size images of spheroids on 3D culture chips on the 5th day. Cancer cells were monocultured or cocultured with ADSCs transfected with siCon, siCCR2, or siPPARα at a 10:1 ratio. The spheroid roundness was calculated as described. Scale bar = 200 µm. Mean ± SD (n = 3); ^*^, *p* < 0.05; ^**^, *p* < 0.001. C) Using a FA quantification kit, we measured the FA concentration in CM obtained from the experiment shown in Figure 5B. Mean ± SD (n = 3); ^*^, *p* < 0.05; ^**^, *p* < 0.001. D) CM collected from the experiment shown in Figure 5B were added to the lower chamber of a transwell system to evaluate its effect on cancer cell migration. After 48 h, the number of migrated cells was determined by counting. Scale bar = 200 µm. Mean ± SD (n = 3); ^*^, *p* < 0.05; ^**^, *p* < 0.001. E) Single‐size images of cancer cells on 3D culture chips on the 5th day. CM obtained from the chips in Figure 5B were applied to 3D culture chips seeded with equal numbers of cancer cells. The treated CM were replaced with newly collected CM every 48 h. The average diameter was measured, and the spheroid roundness was calculated as described. Scale bar = 200 µm. Mean ± SD (n = 3); ^*^, *p* < 0.05; ^**^, *p* < 0.001. F) CM obtained from Figure 5B were applied to 3D culture chips seeded with equal numbers of cancer cells. On day 5, spheroids were collected, sectioned, and mounted on slides. Immunofluorescence (IF) was performed on each slide using the indicated Ki67 antibody and DAPI to assess Ki67 expression levels. Scale bar = 200 µm. Mean ± SD (n = 3); ^*^, *p* < 0.05. G) Cancer cells were monocultured or indirectly cocultured with ADSCs at a 5:1 ratio. Cancer cells were seeded in the bottom wells of a 12‐well transwell plate, and ADSCs transfected with siCon, siCCR2, or siPPARα were cultured in the upper inserts to assess the proliferative effect of indirect coculture. The number of cancer cells in the lower chamber was measured by cell counting following three days of indirect coculture. Mean ± SD (n = 3); ^*^, *p* < 0.05.

Our results demonstrate that cancer‐derived CCL2 acts as a signaling molecule that regulates lipid metabolism in adipose tissues through the CCL2/CCR2/PPARα pathway. We also investigated whether blocking this pathway in ADSCs prior to coculture with cancer cells would alter FA concentrations in the CM and ultimately affect cancer progression. To do this, we transfected ADSCs with siCon, siCCR2, or siPPARα to inhibit CCL2‐derived lipolysis (Figure S5A,B, Supporting Information). Subsequently, we cocultured cancer cells with each group of transfected ADSCs in 3D culture chips to assess changes in cancer progression, as illustrated in the schematic diagram in **Figure** **5**A. When cancer cells were cocultured with ADSCs in which CCL2‐induced lipolysis had been inhibited, their migration was significantly reduced compared to coculture with control ADSCs (Figure 5B and S5C). Additionally, we observed higher FA levels in the CM when cancer cells were cocultured with control ADSCs. However, it was reversed when the CCL2/CCR2/PPARα pathway was inhibited in ADSCs, showing the reduced FA release into the CM (Figure 5C). These findings demonstrate that blocking CCL2‐induced lipolysis in ADSCs successfully disrupted their interaction with cancer cells. In a transwell assay, CM samples from the coculture of cancer cells and control ADSCs significantly enhanced cancer cell migration compared to CM from monoculture, confirming that ADSC‐derived FAs promote this migration. However, the migratory effect was reduced by CM collected from the coculture of cancer cells and ADSCs in which CCL2‐induced lipolysis was inhibited (Figure 5D). Additionally, when equal numbers of cancer cells were seeded into 3D culture chips and treated with the corresponding CM (Figure S5D, Supporting Information), spheroid roundness reflected similar migratory patterns, aligning with the transwell assay findings (Figure 5E, left). Here, the average diameter of spheroids was also measured to assess the proliferative effect of FAs in the CM. The size of cancer cell spheroids was larger in response to CM from coculture of cancer cells with control ADSCs compared to CM from monoculture, indicating enhanced proliferation. Conversely, the CM from cocultures with CCR2‐ or PPARα‐silenced ADSCs resulted in smaller spheroids, reflecting reduced proliferation (Figure 5E, right). After measurement, the spheroids were collected from the 3D culture chips shown in Figure 5E and sectioned onto slides. Immunofluorescence (IF) staining was performed to evaluate the proliferation marker Ki67. Treatment with CM from coculture with control ADSCs, which contained elevated FA levels as indicated in Figure 5C, led to increased cell proliferation along with upregulated Ki67 expression. In contrast, the CM from coculture with CCR2‐ or PPARα‐knockdown ADSCs resulted in reduced proliferation with diminished Ki67 expression (Figure 5F). Additionally, we conducted indirect coculture experiments to confirm whether inhibiting the CCL2/CCR2/PPARα axis in ADSCs affects cancer cell proliferation. As illustrated in Figure S5E (Supporting Information), we seeded equal numbers of cancer cells in the lower chamber of a transwell system. The upper chamber was left empty for monoculture or seeded with ADSCs transfected with siCon, siCCR2, or siPPARα for coculture. After three days of mono‐ or coculture, the number of cancer cells in the lower chamber was quantified. The results showed that indirect coculture with control ADSCs led to a 1.5‐fold increase in the number of cancer cells compared to monoculture. However, this increase was significantly diminished when cancer cells were cocultured with CCR2‐ or PPARα‐silenced ADSCs (Figure 5G). These findings suggest that tumors exploit FAs from neighboring adipose tissues: cancer cell‐derived CCL2 promotes adipose lipolysis and FA release via the CCL2/CCR2/PPARα axis.

### 2.6 Blocking CCL2 Diminishes Adipose Lipolysis, Attenuating Cancer Progression In Vivo

**Figure 6:**
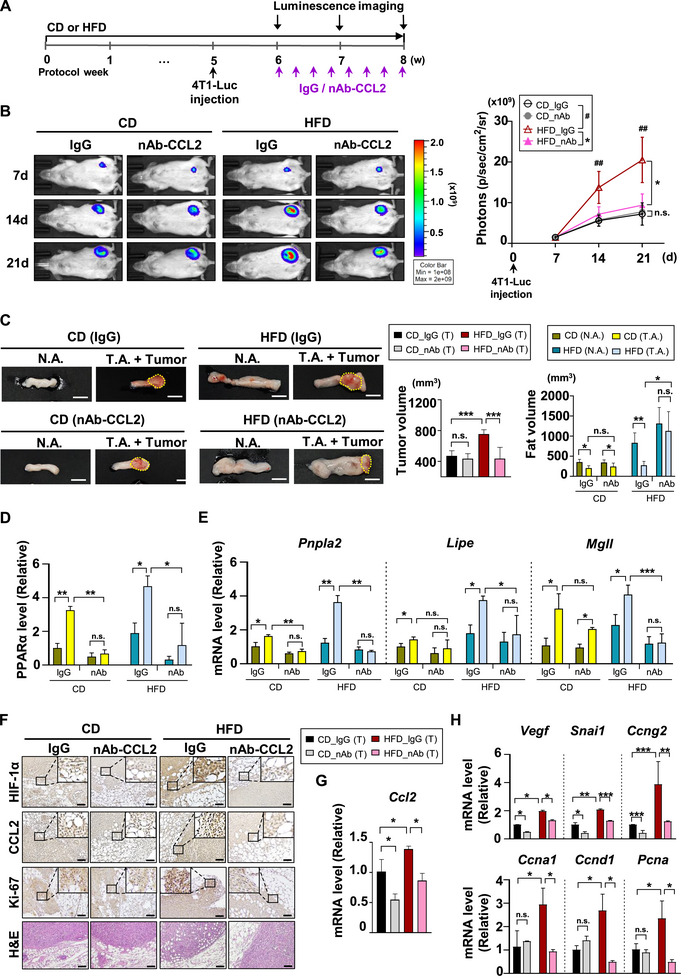
CCL2 Inhibition Decreases Adipose Lipolysis, Attenuating Cancer Progression In Vivo. A) Schematic diagram of the in vivo model. Starting from 4 weeks of age, all the mice were fed either a CD or HFD. At week 5 of feeding, 4T1‐Luc cells were orthotopically injected into the fourth mammary fat pad of female NOD/SCID mice. Starting one week after cancer cell transplantation, nAb‐CCL2 was injected intratumorally every other (n=6 independent animals in each group). B) Bioluminescence images of mice obtained every 7 days after transplantation using the Xenogen IVIS Lumina Spectrum. Color scale bars represent luminescence intensity ranging from low (purple) to high (red). Total flux (photons/sec/cm^2^/sr) was measured and the bioluminescence intensities were plotted. Mean ± SD (n = 6 independent animals in each group); ^*^, *p* < 0.05 (HFD_IgG compared with HFD_nAb‐CCL2); n.s., not significant (CD_IgG compared with CD_nAb‐CCL2); **##**, P < 0.001 (HFD_IgG compared with CD_IgG). C) Representative images of N.A. and T.A. with tumor obtained from IgG injected CD group (CD_IgG), nAb‐CCL2 injected CD group (CD_nAb), IgG injected HFD group (HFD_IgG), and nAb‐CCL2 injected HFD group (HFD_nAb). Each tumor was collected along with the adjacent T.A. The outlines of each tumor were indicated by a yellow dashed line. Scale bar = 10 mm. Tumor and fat volumes (mm^3^) were calculated using the formula: (width × length^2^)/2. Mean ± SD (n =6 in each group); ^*^, *p* < 0.05; ^**^, *p* < 0.001; ^***^, P < 0.0001; n.s., not significant. D) PPARα protein levels in adipose tissues were quantified for each group. Adipose tissue lysates were subjected to western blotting using the indicated antibodies. Band intensities on the blots were analyzed using ImageJ. Mean ± SD (n =6 in each group); ^*^, *p* < 0.05; ^**^, *p* < 0.001; ^***^, *p* < 0.0001; n.s., not significant. E) qRT‐PCR was performed on adipose tissues to assess the expression of lipolysis‐related genes that are downstream targets of PPARα. Mean ± SD (n=6 in each group); ^*^, *p* < 0.05; ^**^, *p* < 0.001; ^***^, *p* < 0.0001; n.s., not significant. F) H&E staining and IHC of tumor sections using the indicated antibodies and DAB staining. Scale bar = 100 µm. (n=6 in each group). G) qRT‐PCR was performed on tumor tissues to assess mRNA level of CCL2. Mean ± SD (n =6 in each group); ^*^, *p* < 0.05. H) qRT‐PCR was performed on tumor tissues to assess the expression of proliferation‐related genes, including HIF‐1α downstream targets, in tumor tissues. Mean ± SD (n=6 in each group); ^*^, *p* < 0.05; ^**^, *p* < 0.001; ^***^, *p* < 0.0001; n.s., not significant.

To see if blocking the crosstalk between adipose tissue and tumors with a neutralizing antibody against CCL2 can suppress cancer progression, we conducted in vivo experiments and analyzed key genes and proteins involved in their interaction. As shown in **Figure** **6**A, NOD/SCID mice were divided into two groups and fed either a CD or HFD. We generated stable 4T1‐Luc cells expressing luciferase to monitor bioluminescence and assess tumor growth. At week 5, when significant body weight difference was noted between the CD and HFD groups (Figure S6A, Supporting Information), all mice received an equal injection of 4T1‐Luc cells. One week later, each group was further divided into two subgroups and treated with either control IgG or nAb‐CCL2 via intra‐tumoral injection every other day. Following the orthotopic injection of 4T1‐Luc cells, tumor growth was monitored weekly, revealing greater growth in the HFD group than in the CD group under control IgG treatment. In the HFD group, nAb‐CCL2 treatment significantly reduced tumor growth compared to the control IgG subgroup, while no such reduction was seen in the CD group (Figure 6B; Figure S6B, Supporting Information). Direct measurements of tumor volume at the time of sacrifice confirmed these results (Figure 6C; Figure S6C, Supporting Information). To thoroughly investigate the alterations in adipose tissues, we meticulously collected tumor‐adjacent adipose tissue alongside the tumor (designated as T.A. + Tumor) as well as non‐tumor adipose tissue (N.A.) from the contralateral side, which is devoid of any tumors. This comprehensive approach allowed us to gain deeper insights into the impact of tumors on neighboring adipose environments. In the HFD group treated with control IgG, T.A. volume significantly decreased compared to N.A. However, with nAb‐CCL2 injection, T.A. volume remained similar to N.A., indicating preservation of adipose tissue from CCL2‐induced lipolysis (Figure 6C; Figure S6C, Supporting Information). The results show that the tumor promotes lipolysis in surrounding adipose tissue by secreting CCL2, which reduces fat pad volume. The lack of significant difference between N.A. and T.A. tissues following CCL2 neutralizing antibody treatment suggests that blocking CCL2 inhibits tumor‐induced lipolysis, supporting earlier in vitro findings. PPARα protein levels were higher in T.A. than in N.A. in IgG‐treated groups, but this increase was not significant within the groups treated with nAb‐CCL2 (Figure 6D; Figure S6D, Supporting Information). Additionally, mRNA levels of lipolysis‐related genes such as *Pnpla2*, *Lipe*, and *Mgll* were elevated in T.A. compared to N.A. in IgG‐treated groups. However, the increase was not observed in the nAb‐CCL2‐treated groups, except for *Mgll* in the CD group. In this group, only *Pnpla2* expression in T.A. was lower with nAb‐CCL2 than with IgG. However, in the HFD group, nAb‐CCL2 treatment reduced the expression of all three lipolysis‐related genes in T.A. compared to IgG treatment. The effect of nAb‐CCL2 on adipose lipolysis was more pronounced under HFD conditions than CD (Figure 6E). These findings demonstrate that tumor‐derived CCL2 enhances lipolysis in adjacent adipose tissue, thereby accelerating cancer progression by increasing FA supply. In contrast, nAb‐CCL2 treatment inhibited lipolysis, preserving adipose tissue volume and suppressing tumor progression by reducing FA supply. Furthermore, tumor tissue analysis showed that CCL2 and HIF‐1α expression levels were higher in the HFD group than in the CD group under IgG treatment, highlighting the role of the FA/HIF‐1α/CCL2 axis in cancer. However, nAb‐CCL2 treatment significantly reduced the levels of CCL2, HIF‐1α, and the proliferation marker Ki‐67, with the effect being particularly evident in the HFD group (Figure 6F,G). Furthermore, genes associated with cell proliferation and survival, which were elevated by HFD, were significantly decreased after nAb‐CCL2 treatment in the HFD group. The HIF‐1α target genes *Vegf, Snai1*, and *Ccng2* showed reduced expression upon nAb‐CCL2 treatment in the CD group, although this did not lead to significant tumor suppression (Figure 6H). Taken together, these findings suggest that obesity‐induced lipid supply increases HIF‐1α expression in tumors, enhancing CCL2 secretion. This process promotes lipolysis and the release of FAs from surrounding adipose tissue, ultimately accelerating tumor progression.

### 2.7 Activation of the HIF‐1α/CCL2/PPARα Axis in Obese Breast Cancer Patients with Tumor Progression and Adipolysis

**Figure 7:**
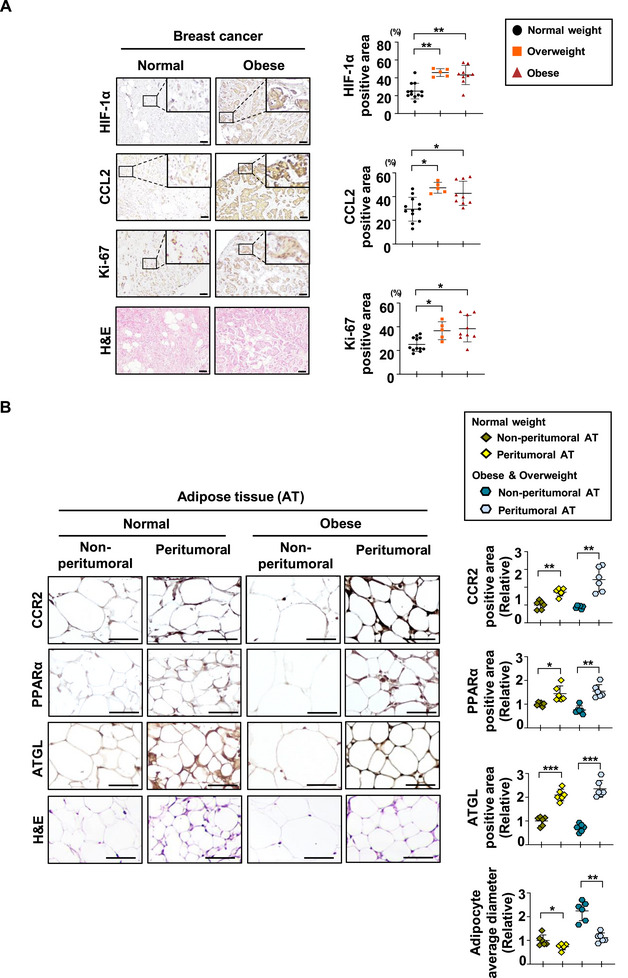
Activation of the HIF‐1α/CCL2/PPARα Axis in Obese Breast Cancer Patients with Tumor Progression and Adipolysis. A) H&E staining and IHC of tumor sections using the indicated antibodies and DAB staining. Total 26 breast cancer patients were stratified into normal, overweight, and obese groups based on their BMI as follows: Normal (20‐22.9 kg m^−2^, n=12; black), Overweight (23‐24.9 kg m^−2^, n=5; orange), Obese (≥25 kg m^−2^, n=9; red). Quantification of the stained areas for HIF‐1α, CCL2, and Ki67 was performed using ImageJ, and the results were visualized as graphs. Scale bar = 100 µm. Mean ± SD; ^*^, P < 0.05; ^**^, P < 0.001. B) H&E staining and IHC of adipose tissue sections using the indicated antibodies and DAB staining. Among the breast cancer patients shown in Figure 7A, 12 individuals with identifiable adipose tissues adjacent to normal breast tissue or tumor tissue were stratified into two BMI‐based groups: Normal (20–22.9 kg m^−^^2^, n = 6; dark yellow and yellow) and Overweight & Obese (≥23 kg m^−^^2^, n = 6; blue and light blue). Quantification of CCR2, ATGL, and PPARα staining per adipocyte was performed using ImageJ. Cytoplasmic staining areas were measured for CCR2 and ATGL, whereas nuclear staining intensity was measured for PPARα. The results are presented as graphs. Scale bar = 50 µm. Mean ± SD; ^*^, *p* < 0.05; ^**^, *p* < 0.001; ^***^, *p* < 0.0001.

We analyzed the expressions of the FA/HIF‐1α/CCL2 axis in breast cancer tissues from patients. We categorized 26 breast cancer patients into normal, overweight, and obese groups based on their BMI. A consistent pattern emerged, supporting previous findings: the expression levels of HIF‐1α, CCL2, and Ki67 in tumor tissues increased progressively with higher BMI. These results suggest that the FA/HIF‐1α/CCL2 axis plays a crucial role in regulating breast cancer progression, particularly as patient obesity increases (**Figure** **7**A). We conducted an examination of the CCR2/PPARα/ATGL pathway in adipose tissues adjacent to normal breast tissue (non‐peritumoral AT) and those adjacent to breast cancer tissue (peritumoral AT) from the same patients. This comparison included normal‐weight individuals as well as overweight and obese groups. The findings revealed that the expression levels of CCR2, PPARα, and ATGL were higher in peritumoral AT than in non‐peritumoral AT, with these differences being more pronounced in the obese group. The average diameter of adipocytes, which indicates the size of lipid droplets within these cells, was found to be smaller in peritumoral AT compared to non‐peritumoral AT. This reduction was even more pronounced in obese breast cancer patients (Figure 7B). These results suggest that cancer‐derived CCL2 activates the CCR2/PPARα pathway in the adipose tissue adjacent to breast cancer, promoting lipolysis and decreasing the size of intracellular lipid droplets. This aligns with the pathway we previously established. Taken together, our findings indicate that in breast cancer patients, obesity enhances the activation of the FA/HIF‐1α/CCL2 axis in tumor tissues and the CCL2/CCR2/PPARα axis in adjacent adipose tissue. This activation accelerates tumor progression and stimulates adipolysis in the adjacent adipose tissue.

### 2.8 A Diagram Illustrating Tumor–Adipose Metabolic Interaction in the Tumor Microenvironment of Obesity

**Figure 8:**
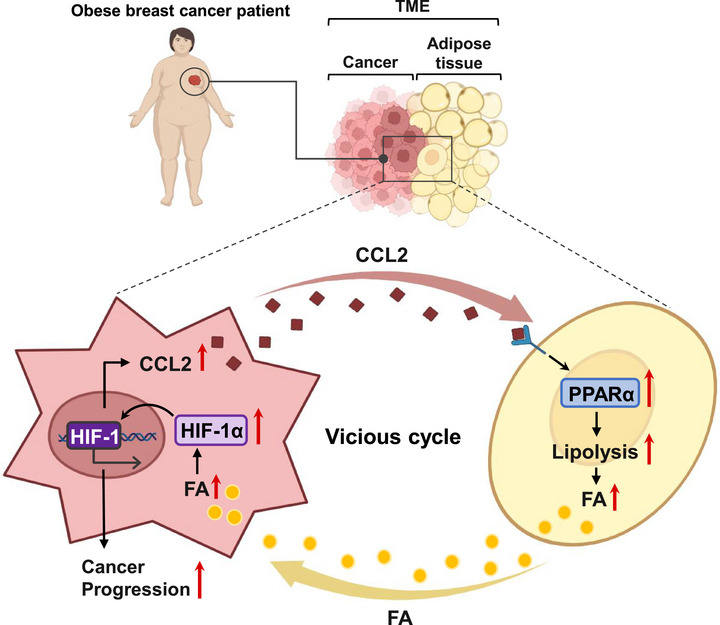
A Diagram of the obesity‐driven tumor progression through tumor–adipose metabolic crosstalk. FA supply activates HIF‐1α in cancer cells, promoting CCL2 secretion. This, in turn, leads to PPARα accumulation and enhances PPARα‐mediated lipolysis in adjacent adipose tissue. Consequently, FAs are continuously released from tumor‐associated adipose tissue, further activating HIF‐1α and amplifying CCL2 production, thereby accelerating tumor progression. This vicious cycle is illustrated in the schematic diagram. TME; Tumor microenvironment, FA; fatty acid.

Obesity leads to an increased supply of lipids, which in turn raises the expression of HIF‐1α and promotes cancer progression. Once activated, HIF‐1α enhances the secretion of CCL2 from the tumor. This secretion then induces lipolysis in the adjacent adipose tissue through the accumulation of PPARα. As a result, the excessive supply of FAs accelerates tumor growth. This excess FA supply accelerates tumor growth, creating a positive feedback loop that worsens tumor progression in obese conditions (**Figure** **8**).

## 3 Discussion

Breast cancer is the most common cancer among women, accounting for ≈ 31% of all cases. In 2020, the World Health Organization (WHO) reported ≈ 2.3 million diagnoses and ≈685000 deaths worldwide due to the disease.^[^ ^28^ ^]^ The global incidence of breast cancer is expected to rise by nearly 40% by 2040, emphasizing the need for improved prevention and treatment.^[^ ^29^ ^]^ Obesity is significantly associated with a higher risk of breast cancer, more aggressive tumor types, and increased mortality rates.^[^ ^30^, ^31^ ^]^ The American Cancer Society's Cancer Prevention Study II reported a strong link between higher body mass index (BMI) and increased breast cancer mortality, consistent with results from Korean patients (Figure 1G).^[^ ^4^ ^]^ Lipid metabolism plays a critical role in maintaining membrane integrity, energy storage, and cellular signaling.^[^ ^32^, ^33^, ^34^ ^]^ Thus, rapidly proliferating cancer cells require a constant supply of lipids for their growth. Our study demonstrates that an environment rich in lipids, often associated with obesity, promotes tumor growth (Figure 1; Figure S2A, Supporting Information). The TME of breast cancer features direct contact between cancer cells and adipose tissue, enabling efficient lipid supply.^[^ ^35^ ^]^ Research indicates that visceral and peritumoral adipose tissues actively promote mammary carcinoma progression by transferring FAs to cancer cells.^[^ ^36^ ^]^ This process is intensified in conditions with increased FA availability like obesity, leading to increased cancer cell proliferation and migration.^[^ ^37^, ^38^ ^]^ In this study, in vivo experiments demonstrated that the adipose tissue adjacent to the tumor was smaller than the contralateral adipose tissue which is not in contact with the tumour. Moreover, the size difference between these two types of adipose tissue was more pronounced under a HFD, which also promoted tumor growth (Figure 1C). These results suggest that FAs derived from adipose tissue enhance cancer cell proliferation, while lipolysis reduces adipose tissue size. As a result, we hypothesize that tumors may trigger lipolysis in the surrounding adipose tissue to obtain the lipids essential for their growth. Our initial objective was to identify the signaling molecules produced by cancer cells that promote lipolysis in nearby adipose tissue.

Hypoxia‐inducible factor‐1 (HIF‐1) plays a crucial role in cellular adaptation to low‐oxygen environments. As a heterodimer composed of an oxygen‐sensitive alpha subunit and a stable beta subunit, HIF‐1 orchestrates a range of responses critical for cell survival. In the hypoxic TME, HIF‐1α undergoes stabilization, triggering the expression of genes that drive angiogenesis, erythropoiesis, metabolism, cell proliferation, and overall survival.^[^ ^39^, ^40^, ^41^, ^42^ ^]^ The overexpression of HIF‐1α across various cancers has become a concerning marker, often correlating with poor patient prognosis.^[^ ^43^, ^44^, ^45^ ^]^ Recent studies have revealed a striking connection between excessive FAs within the TME and the activation of HIF‐1α. Specifically, high levels of FAs stimulate the phosphorylation of AKT and mTOR‐key signaling pathways that enhance HIF‐1α translation.^[^ ^15^, ^16^, ^46^, ^47^ ^]^ Our research has compellingly demonstrated that an increased availability of FAs significantly elevates HIF‐1α expression in cancer cells under obese conditions, both in vivo and in vitro. This elevation is directly linked to the upregulation of several genes associated with cell proliferation. (Figures 1, 2; Figure S2A, Supporting Information). Moreover, HIF‐1α is recognized for its role in regulating metabolic pathways and inflammatory responses as a transcription factor for important cytokines, including CCL2, CCL5, CCL7, IL‐6, and VEGF.^[^ ^18^, ^19^, ^20^, ^21^ ^]^ Building on this foundation, we focused our investigation on identifying the specific cytokines influenced by HIF‐1α in the context of obesity and elevated FA levels. Our comprehensive analysis of cytokine arrays and cocultured cancer cells revealed a significant increase in CCL2 driven by ADSC‐derived FAs among the target cytokines of HIF‐1α (Figure 2D–G). This increase positions CCL2 as a key player in obese condition among the target cytokines influenced by HIF‐1α, highlighting its crucial role in the tumor microenvironment. This insight underscores a critical pathway by which obesity and its metabolic consequences may fuel cancer progression.

CCL2, also known as monocyte chemoattractant protein‐1 (MCP‐1), is a member of the CC chemokine family and located on the q11.2–q12 region of human chromosome 17.^[^ ^48^ ^]^ It encodes a precursor protein of 99 amino acids that is processed into a 75‐amino acid mature form.^[^ ^49^ ^]^ Initially identified as a tumor‐derived chemotactic factor, CCL2 is recognized as a potent chemoattractant for various immune cell populations, including monocytes, natural killer (NK) cells, memory T cells, and immature dendritic cells. This activity contributes to diverse pro‐inflammatory responses and promotes neoangiogenesis.^[^ ^50^ ^]^ The secreted CCL2 exerts its effects by binding to CCR2, one of the 19 known members of the human chemokine receptor family. This interaction activates three distinct signaling pathways: JAK/STAT, MAPK, and PI3K/Akt.^[^ ^24^, ^25^, ^51^ ^]^ The CCL2–CCR2 signaling pathway is implicated in various disorders, including atherosclerosis, multiple sclerosis, asthma, neuropathic pain, diabetic nephropathy, and cancer.^[^ ^52^, ^53^, ^54^, ^55^, ^56^, ^57^, ^58^ ^]^ Our study identifies a novel role for CCL2 in mediating communication between cancer cells and surrounding adipose tissue. In the TME, where tumors are adjacent to adipose tissue, their interaction may involve signaling pathways that influence cancer growth.^[^ ^59^, ^60^, ^61^ ^]^ To effectively replicate the TME that is essential for understanding the crosstalk between tumors and adipose tissue, we employed a cutting‐edge 3D co‐culture chip made of PDMS. This innovative design allows for optimal oxygen supply through the bottom of each well, as previously detailed in our study (Figures 2, 5; Figure S3D, Supporting Information).^[^ ^15^, ^16^ ^]^ We found that CCR2 expression is increased in peritumoral adipose tissue of obese cancer patients, suggesting it is affected by CCL2 from the tumor (Figure 3C; Figure S3H, Supporting Information). This evidence strongly indicates that the FA‐HIF‐1α‐CCL2 axis in cancer plays a pivotal role in activating CCR2‐mediated signaling within the adjacent adipose tissue under obese condition. This interaction highlights the critical link between tumor biology and the metabolic environment, suggesting a significant pathway that could be targeted for therapeutic intervention. We uncovered compelling evidence that CCL2 promotes lipolysis in ADSCs, resulting in reduced intracellular lipid accumulation and increased FAs release into the CM (Figure 3; Figure S3L,M, Supporting Information). This discovery offers new insights into how adipose tissue can profoundly influence cancer growth, highlighting the urgent need for a deeper understanding of this relationship. We found that tumor‐derived CCL2 acitively upregulates PPARα, a key transcription factor in lipid metabolism, thereby promoting adipose lipolysis and the release of FAs (Figure 3; Figure S3K, Supporting Information). This cascade of events reactivates the FA–HIF‐1α–CCL2 axis within the cancer environment. Ultimately, this dynamic interaction between tumor and adipose tissue establishes a self‐perpetuating vicious cycle that fuels cancer progression (Figure 8). Up to this point, the majority of research investigating the link between obesity and cancer has concentrated largely on the role of adipokines released from adipose tissue and their impacts on the immune system.^[^ ^10^, ^62^, ^63^ ^]^ However, our groundbreaking findings shed light on the critical FA‐HIF‐1α‐CCL2 axis in obesity‐related cancer, providing a novel perspective.

Peroxisome proliferator‐activated receptors (PPARs) are ligand‐activated transcription factors within the nuclear receptor superfamily and consist of three well‐characterized subtypes: PPARα (NR1C1), PPARβ/δ (NR1C2), and PPARγ (NR1C3).^[^ ^64^, ^65^, ^66^, ^67^, ^68^ ^]^ Among them, PPARα, the first identified member of this family, plays a pivotal role in regulating a wide array of genes integral to various lipid metabolic pathways.^[^ ^69^, ^70^ ^]^ It is predominantly expressed in key organs including the heart, liver, skeletal muscle, and adipose tissue, where it serves as an essential regulator of FA homeostasis.^[^ ^71^, ^72^, ^73^, ^74^ ^]^ Crucially, PPARα has been shown to mediate lipolysis within adipose tissue.^[^ ^75^, ^76^ ^]^ Both internal and external cellular signals can influence the subcellular localization, stability, and turnover of PPARα, thereby modulating its regulatory effects on cellular functions.^[^ ^27^, ^69^ ^]^ A key finding of our research reveals that PPARα stability can be notably regulated by HUWE1, an E3 ubiquitin ligase, under the influence of cancer‐derived CCL2. Our results indicate that CCL2 activates the p‐ERK signalling pathway, enhancing the stability of PPARα at the protein level in adipose tissue by diminishing its interaction with HUWE1 (Figure 4). Overall, CCL2‐induced activation of PPARα in adipose tissue enhances lipolysis and promotes FA release into the TME, facilitating lipid supply to adjacent tumors. This dynamic interaction between adipose tissue and the tumor accelerates tumor growth in the context of obesity, as cancer cells exploit adjacent adipose tissue to meet their heightened lipid demands. Thus, our study elucidates the underlying mechanism, providing insights into how obesity can accelerate cancer progression.

Numerous studies have shown that cytokines are pivotal drivers of disease progression in cancer patients, leading to the development of innovative therapeutic strategies that block cytokine‐related signaling pathways.^[^ ^77^, ^78^, ^79^, ^80^, ^81^, ^82^ ^]^ In this study, we explored three powerful approaches to disrupt the reciprocal signaling loop between cancer cells and ADSCs in coculture: 1) silencing HIF‐1α and CCL2 in cancer cells, 2) silencing CCR2 and PPARα in ADSCs, 3) treatment with nAb‐CCL2. Each of these methods effectively reduced the levels of ADSC‐derived FAs in CM of coculture. Furthermore, treatment of cancer cells with the CM significantly hindered cancer progression, as illustrated in Figure 3A,B, and Figure S3B,F,G, and 5. Our in vivo experiments demonstrated that disrupting the tumor‐adipose tissue feedback loop with nAb‐CCL2 not only inhibited tumor growth but also decreased lipolysis in tumor‐adjacent adipose tissue, thereby preserving its volume in the HFD group. This potent inhibitory effect of nAb‐CCL2 was supported by expression analyses of genes involved in the feedback loop (Figure 6). As previously mentioned, CCL2 has the potential to influence immune cells. To eliminate immune‐mediated effects during the administration of nAb‐CCL2, we utilized NOD/SCID mice, which lack functional T and B lymphocytes and have severely impaired NK cells and other immune activities. In our study, the dose and frequency of nAb‐CCL2 injections significantly suppressed tumor growth in the HFD group but not in the normal diet group. This suggests that the observed tumor‐suppressive effect was primarily due to changes in lipid metabolism rather than immune activity. Additionally, since CCL2 has been reported to predominantly recruit M2‐like macrophages,^[^ ^83^ ^]^ we conducted immunohistochemical staining for the M2 marker CD206. The results showed no significant differences between the treated and untreated groups (Figure S6E, Supporting Information). This indicates that the extent of nAb‐CCL2 administration in this study was insufficient to affect M2 macrophage infiltration into tumors in immunodeficient mice. In conclusion, these experimental conditions confirm that the observed inhibition of tumor growth by nAb‐CCL2 resulted from regulation of lipid metabolism rather than immune modulation. The reciprocal interaction between cancer and adipose tissue was compellingly validated in breast cancer patients. The human data shows that obesity enhances the activation of the FA/HIF‐1α/CCL2 pathway in tumor tissue and the CCL2/CCR2/PPARα axis in adjacent adipose tissue, thereby accelerating tumor progression and promoting adipolysis in the surrounding adipose tissue (Figure 7). These findings suggest that the crosstalk between cancer and adipose tissue becomes more pronounced in an obese environment.

In obese conditions, adipose tissue accumulates excess FAs that are enzymatically esterified with glycerol to form diglycerides (DG) and triglycerides (TG). This process leads to the expansion and enlargement of lipid droplets.^[^ ^84^ ^]^ These enlarged lipid reservoirs enable CCL2‐driven adipolysis, resulting in a significantly greater release of FAs into the TME, thereby promoting tumor growth. Consequently, blocking CCL2 with a nAb‐CCL2 more effectively limits the supply of FAs to tumors in an obese state, thereby exerting a stronger tumor‐suppressive effect in obesity‐associated breast cancer. Therefore, the therapeutic impact of nAb‐CCL2 is expected to be particularly beneficial for patients with breast cancer who are obese. Similarly, prostate cancer also has a close interaction with adipose tissue. Specifically, the periprostatic adipose tissue (PPAT), which surrounds the prostate, plays a crucial role in its TME.^[^ ^85^ ^]^ The same in vitro experiments conducted with the prostate cancer cell line PC3 demonstrated similar findings. These observations suggest that CCL2 could be a promising therapeutic target not only for obesity‐related breast cancer but also for other cancers associated with adipose tissue, such as prostate and colon cancer.

In summary, our study establishes a critical interplay between two axes: the FA–HIF‐1α–CCL2 axis in cancer and the CCL2–PPARα–FA axis in adipose tissue. FAs released from adipose tissue activate HIF‐1α in cancer cells, which in turn increases the secretion of CCL2, promoting tumor growth and metastasis. In turn, CCL2 produced by tumors triggers PPARα‐mediated lipolysis in adjacent adipose tissue, leading to the further release of FAs that can affect the tumors again. This reciprocal interaction is augmented in individuals with obesity, where the adipose tissue surrounding tumors accumulates larger stores of FAs and consequently provides a greater supply of these lipids. This increased availability of lipids has a more pronounced effect on tumor progression, leading to a stronger tumor‐suppressive response when CCL2 is blocked. Therefore, this study clarifies the relationship between obesity and cancer, highlighting CCL2 as a promising therapeutic target for cancer patients who are obese.

## 4 Experimental Section

### 2D Cell Culture

4T1 cells (RRID: CVCL_0125) were obtained from the American Type Culture Collection (ATCC) (Manassas, VA, USA) and cultured in Dulbecco's modified Eagle's medium (DMEM) (Welgene) supplemented with 10% fetal bovine serum (FBS) (Welgene) and 1% penicillin/streptomycin (Thermo, Rockford, IL, USA) at 37 °C and 5% CO_2_. MDA‐MB‐231 (RRID: CVCL_0062), MCF7 (RRID: CVCL_0031), and PC3 (RRID: CVCL_0035) cells were purchased from the Korea Cell Line Bank (KCLB) (Seoul, Republic of Korea) and cultured in Roswell Park Memorial Institute 1640 medium (RPMI1640) (Welgene) supplemented with 10% FBS (Welgene) and 1% penicillin/streptomycin (Thermo, Rockford, IL, USA) at 37 °C and 5% CO_2_. All cell lines were confirmed to be free of any contamination.

### Isolation and Culture of Adipose‐Derived Stem Cells

The isolation of adipose‐derived stem cells (ADSCs) was performed as previously reported.^[^ ^86^ ^]^ Human adipose‐derived stem cells (hADSCs) were isolated by mincing human adipose tissues and enzymatically digesting them in Hanks’ Balanced Salt Solution (HBSS; Sigma‐Aldrich, St. Louis, MO, USA) containing 0.2% collagenase type I (Worthington Biochemical Corporation, Lakewood, NJ, USA) for 1 h at 37 °C. This was conducted with the approval of the Institutional Review Board of Seoul National University Hospital (Approval No. H‐1602‐110‐742). All the experimental procedures adhered to the guidelines established by the Institutional Review Board of Seoul National University Hospital. Following the inactivation of collagenase activity, the resulting cell suspension was filtered through a 40 µm cell strainer (BD Biosciences, San Jose, CA, USA). The suspension was then centrifuged at 420 × g for 5 min to remove floating adipocytes, after which the stromal vascular fraction (SVF) cells were collected. The hADSCs were isolated through successive subculturing in DMEM containing low glucose and pyruvate (Thermo Fisher Scientific), supplemented with 10% FBS and 1% penicillin‐streptomycin. In case of mouse adipose‐derived stem cells (mADSCs) isolation, the mouse adipose tissues were collected from fourth mammary fat pads of eight‐week‐old female BALB/c from KOATECH (Pyeongtaek, Korea). This was performed by the guidelines of the Seoul National University Institutional Animal Care and Use Committee (approval No. SNU‐230913‐3‐1). After mincing and enzymatically digesting them in Hanks’ Balanced Salt Solution (HBSS; Sigma‐Aldrich, St. Louis, MO, USA) containing 0.2% collagenase type I (Worthington Biochemical Corporation, Lakewood, NJ, USA) for 30 min at 37 °C, the cell suspension was filtered through a 40 µm cell strainer. Then it was centrifuged at 420 × g for 5 min to collect SVF cells. Then the mADSCs were isolated through repeated subculturing in DMEM/F‐12 (Thermo Fisher Scientific), supplemented with 10% FBS and 1% penicillin‐streptomycin. All the cells were incubated at 37 °C with a humidified atmosphere containing 5% CO2 and 21% O2.

### Small Interfering RNAs and Plasmids Transfection

All siRNAs were synthesized by Integrated DNA Technologies (Coralville, IA, USA). Cell transfections were performed using Lipofectamine RNAiMAX, following the manufacturer's protocol (Thermo Fisher Scientific, Newark, DE, USA). The sequences of the siRNAs used in this study are provided in Table S1 (Supporting Information). The PPARA plasmid was purchased from Addgene and transfected into cells using Lipofectamine 2000 (Thermo Fisher Scientific, Newark, DE, USA).

### In Vitro Antibodies and Chemicals

Recombinant Human CCL2/JE/MCP‐1 (Catalogue # 279‐MC, R&D systems) was treated at a concentration of 10 or 20 ng mL^−1^. For neutralizing CCL2, human neutralizing antibody‐CCL2 (#AF‐279‐NA, R&D systems) was treated to human cell lines at a concentration of 1 µg mL^−1^ and mouse neutralizing antibody‐CCL2 (#AB‐479‐NA, R&D systems) was treated to mouse cell lines at a concentration of 20 µg mL^−1^. Both neutralizing antibodies of CCL2 are labelled as nAb‐CCL2 in this study. The information of the antibodies used for immunoblotting in this study is provided in Table S3 (Supporting Information). To inhibit ERK pathway, PD98059 (#HY‐12028, Med Chem Express) was treated at a concentration of 20 µm, and the PPARα antagonist, GW6471, (#ab254317, Abcam) was treated at a concentration of 1 µm. Cells were treated with 2% lipid mixture (LM) (L0288, Sigma) for the indicated experiments. Cells were treated with MG132 (BML‐PI102, Enzo Life Sciences, Plymouth Meeting, PA, USA) at a concentration of 10 µm and cycloheximide (CHX) (C1988, Sigma‐Aldrich) at a concentration of 100 µM.

### PDMS 3D Culture System

Oxygen‐permeable spheroid culture devices were designed by Prof. Fukuda.^[^ ^87^, ^88^ ^]^ The PDMS chips were formed as it had been mentioned in the previous article.^[^ ^15^ ^]^ A total of 5 × 10^5^ cells (only cancer cells or with ADSCs in 10:1 ratio) was dispersed in 3 mL of culture medium and seeded onto the PDMS chips pre‐coated with 4% pluronic solution (Sigma) for 16 h to prevent cell attachment during culture. The cells were then cultured on the PDMS chips for 5 days, and the average diameters of the resulting spheroids were analyzed using ImageJ. Spheroid roundness was calculated as follows: roundness (%) = 100–(R–r)/R^*^100, where R represents the radius of the maximum circumscribed circle and r represents the minimum inscribed concentric circle.^[^ ^89^ ^]^

### Immunofluorescence for Sectioned 3D Culture Spheroids

Immunofluorescence staining was performed on frozen sections of the cells' 3D spheroids. Spheroids collected from each plate were fixed with 4% paraformaldehyde (Biosesang, Gyeonggi‐do, South Korea) for 30 min at 4 °C and washed three times with PBS. They were then sequentially immersed in 10%, 20%, and 30% sucrose solutions for 1 h each. After embedding in OCT compound (Sakura Finetek, Tokyo, Japan) and storing at −80 °C for 24 h, the OCT blocks were sectioned into 10 µm‐thick slices and mounted on glass slides. Then the mounted sections were washed with PBS and blocked with 1% BSA solution for 1. Following the blocking step, they were incubated overnight at 4 °C with Ki‐67 primary antibody. After three washes with 0.1% Tween‐20 in PBS (PBST), the sections were incubated with Alexa Fluor 568‐conjugated anti‐rabbit secondary antibody (1:400 dilution) for 1 at room temperature. After three additional washes with PBST, the nuclei were counterstained with DAPI (1:400 dilution) for 5 min. Immunofluorescence images were then acquired with a confocal microscope (Olympus, Tokyo, Japan).

### Sorting of GFP Stable Cells from 3D Co‐Culture System

4T1, MDA‐MB‐231, MCF7, and PC3 cells were transfected with GFP plasmid. The cells stably expressing the plasmid were selected with G418 (Sigma) and sorted with a flow cytometer (BD FACS Aria II, BD Biosciences, San Jose, CA, USA). Then the GFP tagged stable cancer cells were cultured on the 3D culture chips alone or with ADSCs in 10:1 ratio for 3 days. After that, the cells were collected from 3D culture chips and only GFP tagged cancer cells were sorted with the flow cytometer.

### Transwell Migration Assay

Transwell migration assays were performed using Transwell inserts (6.5 mm diameter, 8 µm pore size; Corning Inc., Corning, NY, USA) pre‐coated with 0.5 mg mL^−1^ collagen. Cells were seeded into the upper chamber in 200 µL of serum‐free medium, while 550 µL of conditioned medium (CM) was added to the lower chamber as a chemoattractant. After 48 h, the cells were fixed with methanol for 3 min and stained with 0.1% crystal violet in 2% methanol for 45 min at room temperature. Non‐migrated cells on the upper surface of the membrane were removed using a cotton swab. Migrated cells on the lower surface of the membrane were visualized in four randomly selected high‐power fields and quantified using ImageJ software.

### Western Blotting and Immunoprecipitation

Protein samples were separated by sodium dodecyl sulfate‐polyacrylamide gel electrophoresis (SDS‐PAGE) and transferred onto Immobilon‐P membranes (Millipore, Bedford, MA, USA). The membranes were then blocked with 5% skim milk in Tris‐buffered saline containing 0.1% Tween 20 (TBST) for 1 h, followed by overnight incubation with the primary antibody at 4 °C. After brief washing with TBST, the membranes were incubated with a horseradish peroxidase (HRP)‐conjugated secondary antibody for 1 h. Protein signals were detected using an ECL Plus kit (Thermo Fisher Scientific). To precipitate tagged proteins, transfected cells were lysed in a buffer containing 5 mm EDTA, 50 mm Tris‐Cl, 100 mm NaCl, 0.1% NP‐40, and a protease inhibitor cocktail (Sigma–Aldrich). Cell lysates were incubated with EZview anti‐HA affinity beads (Sigma‐Aldrich) at 4 °C for 4 h. For the precipitation of endogenous PPARα binding with HUWE1, cell lysates were incubated with an HUWE1 antibody overnight at 4 °C, followed by incubation with Protein A/G Sepharose beads (GE Healthcare Life Sciences, Marlborough, MA, USA) for 4 h at 4 °C. After three times of wash with lysis buffer, the proteins bound to the beads were eluted using 2× SDS denaturing buffer and analyzed by western blotting.

### RNA Isolation and Quantitative Real‐Time PCR (qRT‐PCR)

Total RNA from cultured cells was extracted using TRIzol Reagent (Invitrogen). cDNA synthesis was performed using the Easy Script cDNA Synthesis Kit (Applied Biological Materials Inc., Richmond, Canada). qRT‐PCR was conducted on 96‐well plates using the Eva Green qPCR Master Mix reagent (Applied Biological Materials) in a Step One Real‐Time PCR system (Applied Biosystems, Foster City, CA, USA). Primer sequences for qRT‐PCR are provided in Table S2. The mRNA expression levels of target genes were normalized to 18S rRNA levels.

### Luciferase Assay

The proliferator response element (PPRE)‐ luciferase reporter plasmid was purchased from Addgene (PPRE X3‐TK‐luc, #1015) for PPARα reporter assay. The luciferase reporter plasmid was transfected into ADSCs. After 24 h of stabilization, indicated drugs were treated to the cells for another 24 h. Then luciferase activities were measured using a Lumat LB9507 luminometer (Berthold Technologies, Bad Wildbad, Germany), and the reporter activity was divided by B‐galactosidase activity to normalize transfection efficiency.

### Orthotopic Breast Cancer Mouse Models and In Vivo Luminescence

All the experiments for animal study were performed by the guidelines of the Seoul National University Institutional Animal Care and Use Committee (approval No. SNU‐220406‐5‐4). Four‐week‐old female NOD/SCID mice were purchased from KOATECH (Pyeongtaek, Korea) and housed in a pathogen‐free facility at a temperature of 22–26 °C and humidity of 40–60% under a 12‐h light/12‐h dark cycle. Starting from 4 weeks of age, all the mice were divided into two groups and were fed either a chow diet or a high‐fat diet. Body weight were measured every week. 4T1 cells were transfected with luciferase‐IRES‐GFP plasmid. The cells stably expressing the plasmid were selected with G418 (Sigma) and sorted with a flow cytometer (BD FACS Aria II, BD Biosciences, San Jose, CA, USA). Orthotopic tumors were induced by injecting 2 × 10^5^ 4T1 cells suspended in 50 µL of PBS into the fourth (inguinal) mammary fat pad. Tumor growth was assessed by measuring individual tumors and calculating tumor volume: Tumor volume (mm3) = (width × length^2^)/2. Also, the tumor growth was monitored using the Xenogen IVIS Lumina in vivo imaging technology platform (Xenogen, Alameda, CA). For in vivo luminescence, 100 µL of VivoGlo luciferin (Promega, Madison, WI, USA) in sterilized PBS (40 mg mL^−1^) was intraperitoneally injected into the anaesthetized mice. After 10 min, images were acquired with the Xenogen IVIS Lumina series and assessed using the Living Image 2.11 software package (Xenogen Corp., Waltham, MA, USA).

### In Vivo Treatment of CCL2 Neutralizing Antibody or IgG

A monoclonal antibody neutralizing murine CCL2 (nAb‐CCL2) (InVivoMAb anti‐mouse/human/rat CCL2, clone number: 2H5, #BE0185) was purchased from Bio X Cell (West Lebanon, NH, USA). Starting on day 7 after 4T1 cell inoculation, the nAb‐CCL2 was injected intratumorally every other day for 2 weeks at a concentration of 2.0 mg kg^−1^ per dose in 100 uL of PBS.^[^ ^90^ ^]^ For the isotype control, IgG (InVivoMAb polyclonal Armenian hamster IgG, #BE0091, Bio X Cell) was used at the same concentration.

### Conditioned Media

Conditioned media (CM) from monocultured or cocultured 3D culture chips were collected after 48 h of culturing. Before treatment to cells, the collected CM were centrifuged at 12000 rpm for 20 min to remove debris, and the supernatants were filtered through 0.45 µm Minisart filters (Sigma). To deplete lipids, the CM were incubated with activated charcoal (40 mg mL^−1^, Sigma) for 8 h at 4 °C. Following incubation, the supernatants were collected by centrifugation at 12000 rpm for 20 min and subsequently filtered through 0.2 µm Minisart filters (Sigma).

### Cytokine Array

Cytokine Array kits were used for CM from monocultured or cocultured 3D culture chips, following the manufacturer's protocol (ab133996 for human cell lines, ab133993 for mouse cell lines, Abcam)

### Indirect Coculture System

Indirect Coculture was performed using Transwell inserts (6.5 mm diameter, 0.4 µm pore size; Corning Inc., Corning, NY, USA). Cancer cells were seeded on the bottom of 12‐well plates 24 h before the upper chambers were placed. ADSCs were transfected with each siRNA according to the experimental design 24 h prior to being seeded in the upper chambers. Cancer cells and ADSCs were indirectly cocultured at a 5:1 ratio.

### Fatty Acid Quantification

The Fatty Acid Quantification Kit (MAK044, Sigma) was used for CM according to the manufacturer's protocol.

### Nile Red Staining

After washing with PBS, cells were fixed with 4% paraformaldehyde (PFA) for 10 min at room temperature. After fixation, each well of samples were incubated with Nile red (1 mg mL^−1^) for 20 min at room temperature. The cells were stained with 4′,6‐diamidino‐2‐phenylinodle (DAPI) for 1 min subsequently. The slides were mounted with FluorSave Reagent for visualization under a fluorescence microscope. For flow cytometric analysis, cells were stained with Nile Red solution and DAPI, and then analyzed using a flow cytometer (FACSymphony A5, BD Biosciences).

### Immunohistochemical Analysis (IHC)

Tissue sections were incubated at 60 °C for 1 h and then microwaved in antigen retrieval solution for 20 min. Following treatment with 3% hydrogen peroxide (H_2_O_2_), the slides were incubated with PBS containing 3% BSA and 0.3% Triton X‐100 for 1 h at room temperature. The slides were then incubated overnight at 4 °C with indicated antibodies. After a brief wash, the slides were incubated with a biotinylated secondary antibody (1:200) for 1 h. The immune complexes were visualized using a Vectastain ABC kit (Vector Laboratories, Burlingame, CA, USA). Finally, the tissue slides were counterstained with hematoxylin for 10 min.

### Immunofluorescence Staining (IF)

Cells were fixed with 4% paraformaldehyde for 10 min and permeabilized with PBS containing 0.3% Triton X‐100. After blocking with PBS containing 3% BSA and 0.3% Triton X‐100 for 1 h at room temperature, the cells were incubated overnight at 4 °C with the primary CCL2 antibody at the concentration specified in Table S3 (Supporting Information). Following three washes with PBS, the cells were incubated with a fluorescent‐conjugated secondary antibody (Alexa Fluor 568, Invitrogen) for 1 h at room temperature. Nuclei were stained with DAPI and F‐actin was stained with Alexa Fluor 488 phalloidin (Invitrogen). The slides were mounted using FluorSave (Merck Millipore, Billerica, MA) and the images were captured using a confocal microscope (FV3000; Olympus, Tokyo, Japan).

### Human Breast Tissues for IHC

The super biochips containing breast tumor tissues of 26 breast cancer patients were obtained from Seoul National University Hospital (Seoul, South Korea) with consent under approval from the Institutional Review Board of Seoul National University Hospital (Approval No. H‐2207‐198‐1345). Detailed patient information is presented in Table S4 (Supporting Information).

### Statistical Analysis

All data were analyzed using GraphPad Prism 8 software, and the results are expressed as means ± standard deviation (SD) or standard error of the mean (SEM). To compare differences between groups, a two‐tailed, unpaired Student's t‐test was conducted. A *p*‐value of less than 0.05 was considered statistically significant.

## Conflict of Interest

The authors declare no conflict of interest.

## Author Contributions

J.E.Y., J.E.S., Y.S.S., D.W.J., J.F., J.W.K., J.W.P., and Y.S.C. performed methodology; J.E.Y. and J.E.S. performed acquisition of data; J.E.Y., J.E.S., and Y.S.C. performed analysis of data; J.E.Y. wrote‐original draft; Y.S.C. performed supervision.

## Supporting information



Supporting Information

## Data Availability

The data that support the findings of this study are available on request from the corresponding author. The data are not publicly available due to privacy or ethical restrictions.
