# Supplementary material for: Cancer Manipulates Adjacent Adipose Tissue to Exploit Fatty Acids via HIF‐1α/CCL2/PPARα Axis: A Metabolic Circuit to Support Tumor Progression
Source: Adv Sci (Weinh). 2025 Oct 29;13(3):e15186. doi: 10.1002/advs.202515186 (PMC12806468; doi:10.1002/advs.202515186)
Supplement: Supplementary file 1 — Supporting Information [file ADVS-13-e15186-s001.pdf]

Supplementary Information for

Cancer Manipulates Adjacent Adipose Tissue to Exploit Fatty Acids via HIF-1α/ CCL2/PPARα Axis: A Metabolic Circuit to Support Tumor Progression

*Jeong-Eun Yun, Jieun Seo, Jiwon Koh, Seock-Ah Im, Ki Yong Hong, Yeseon Son, Do-Won Jeong, Junji Fukuda, Jong-Wan Park, and Yang-Sook Chun**

Supplementary Figures .... **(2)**

Supplementary Tables .... **(18)**

Supplementary Figures

Supplementary Fig. 1

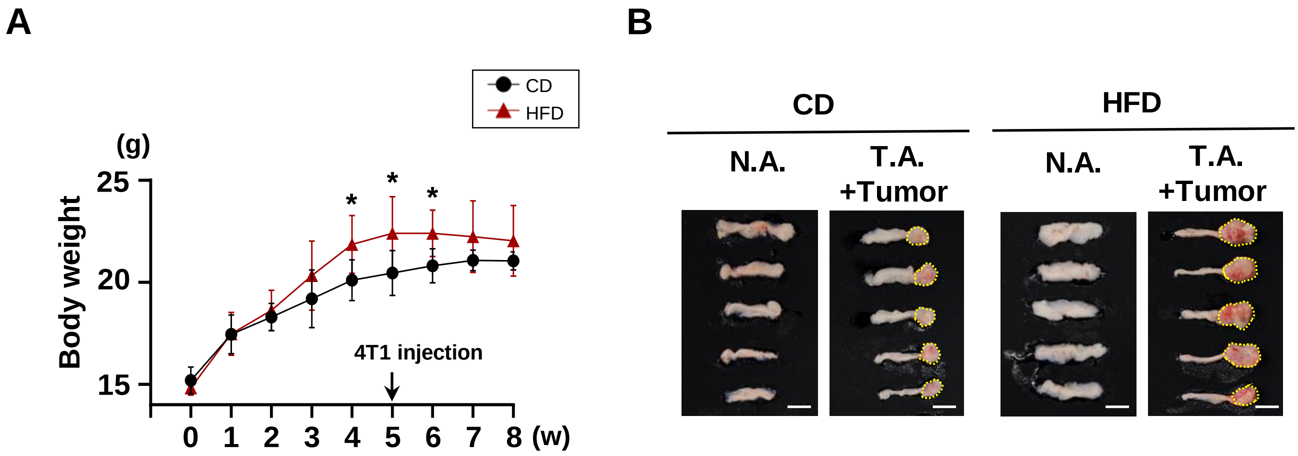

Supplementary Figure 1: Extended data for Fig.1

(A) Body weights were measured every week. Mean ± SD (n =5 in each group); *P < 0.05. (B) Tumors along with the adjacent adipose tissues (tumor-adjacent adipose tissue; T.A.) are from fourth mammary fat pad of right side of mice. Each tumor of mice was collected along with the T.A. The outlines of each tumor are indicated by a yellow dashed line. The contralateral adipose tissues (non-tumor adipose tissue; N.A.) are from fourth mammary fat pad of left side of mice (n = 5 independent animals for each group). Scale bar = 10 mm.

Supplementary Fig. 2

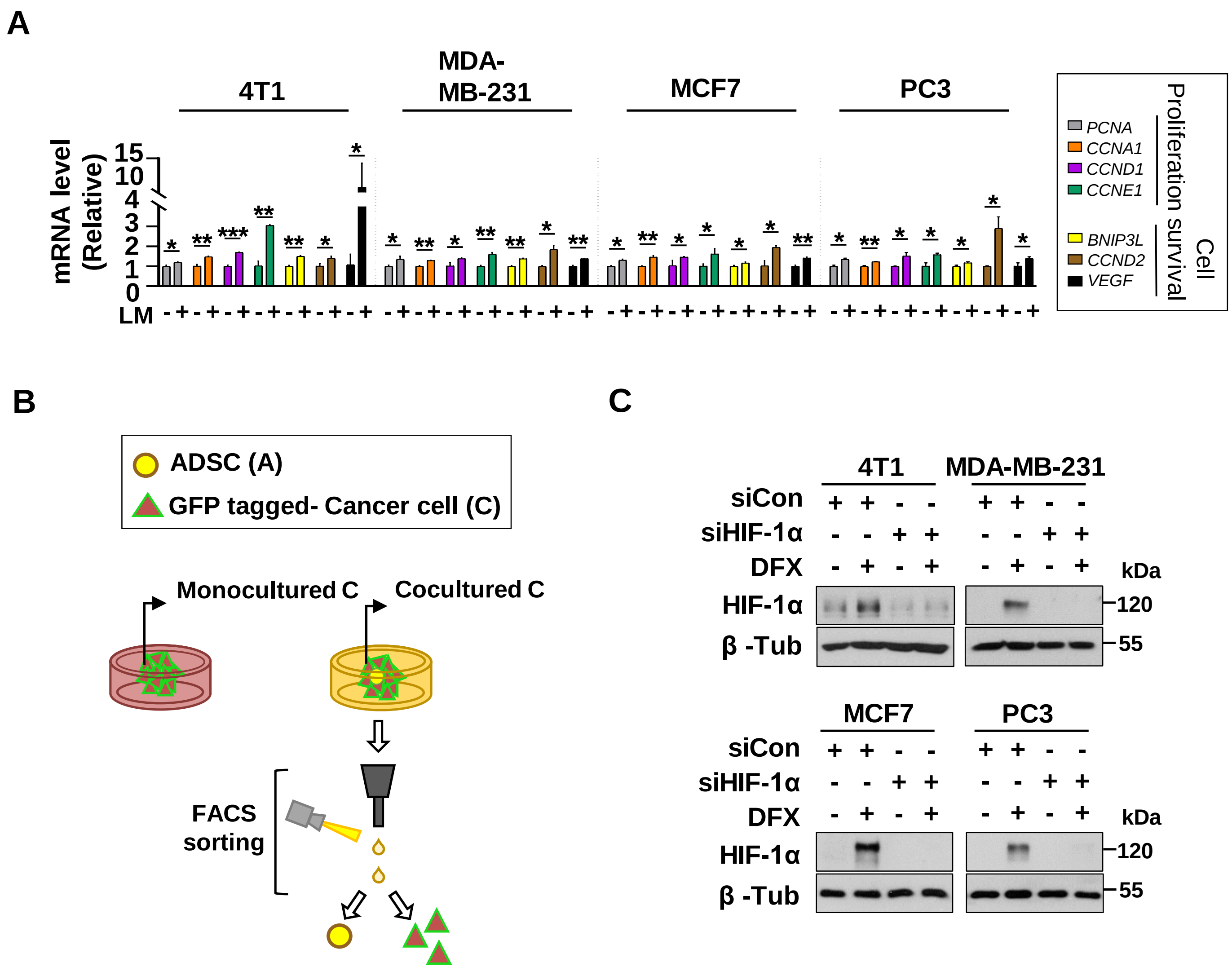

Supplementary Fig. 2: Extended data for Fig.2

(A) The expressions of proliferation and cell survival-related genes, including HIF-1α target genes, were quantified by qRT-PCR relative to 18S RNA after 48-hour treatment with 2% LM to cancer cells. Mean ± SD (n = 3); *, P < 0.05; **, P < 0.001. (B) Schematic diagram of fluorescence-activated cell sorting (FACS) for collecting cocultured GFP tagged-cancer cells. GFP tagged cancer cells were monocultured or cocultured with ADSCs for 3 days and sorted with a flow cytometer. (C) After transfection with siCon or siHIF-1α, the cancer cells were treated with deferoxamine (DFX) (130μM) for 8 hours. The cancer cells were subjected to western blotting to confirm that siHIF-1α effectively knocked down HIF-1α expression.

Supplementary Fig. 3

A

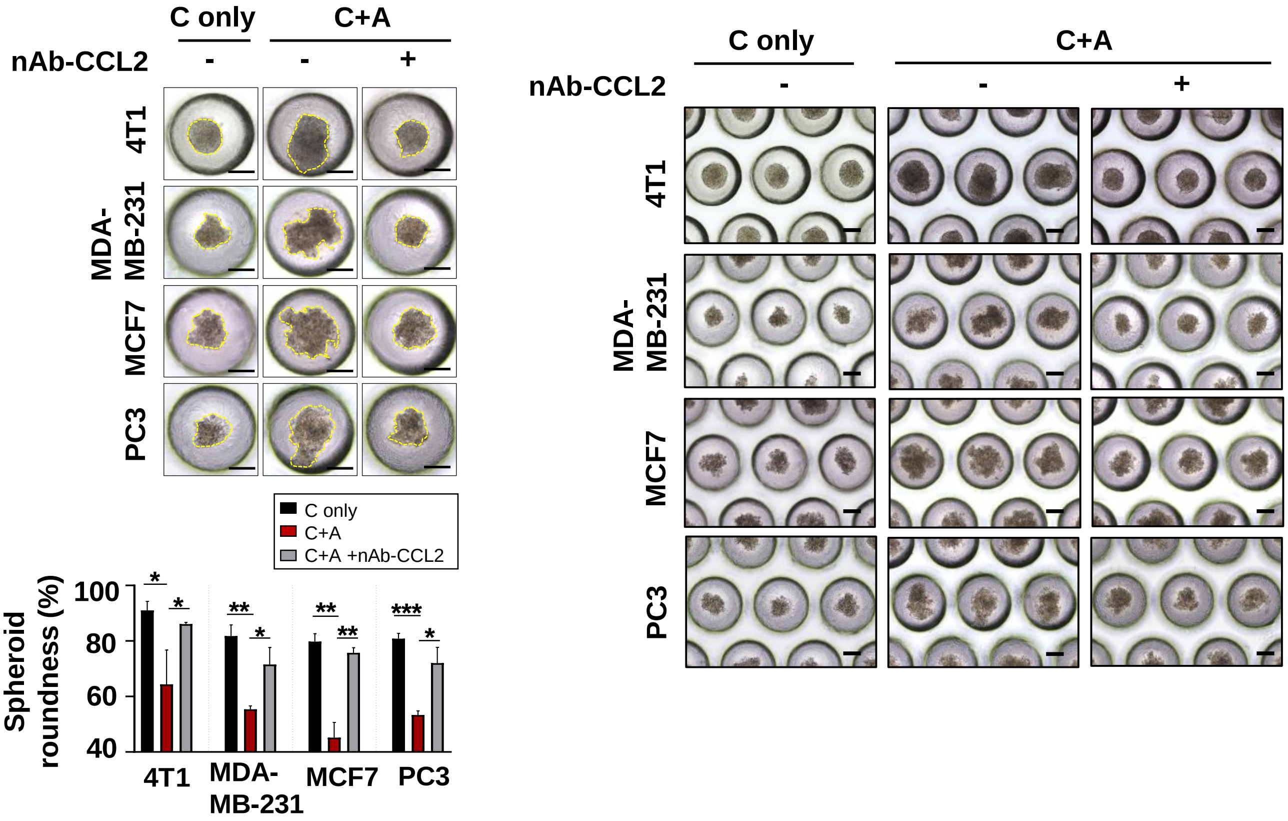

B

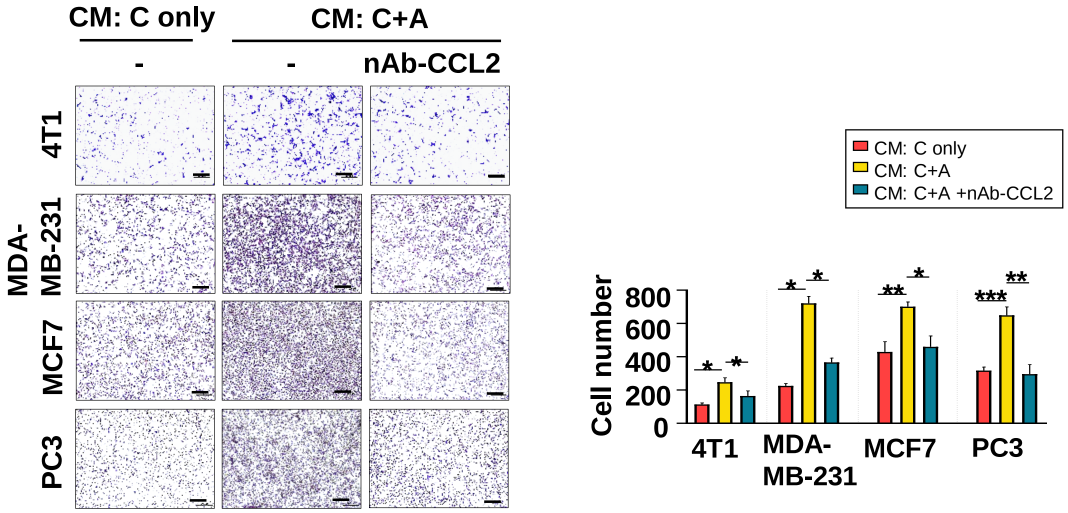

Supplementary Fig. 3 (Continue)

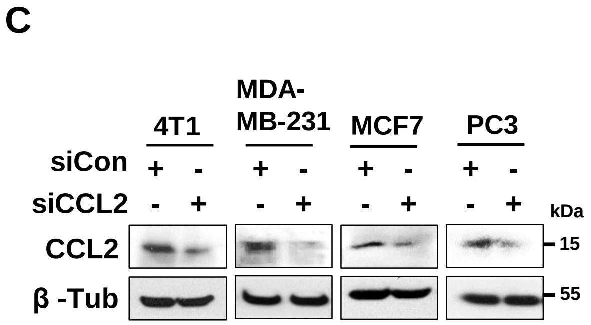

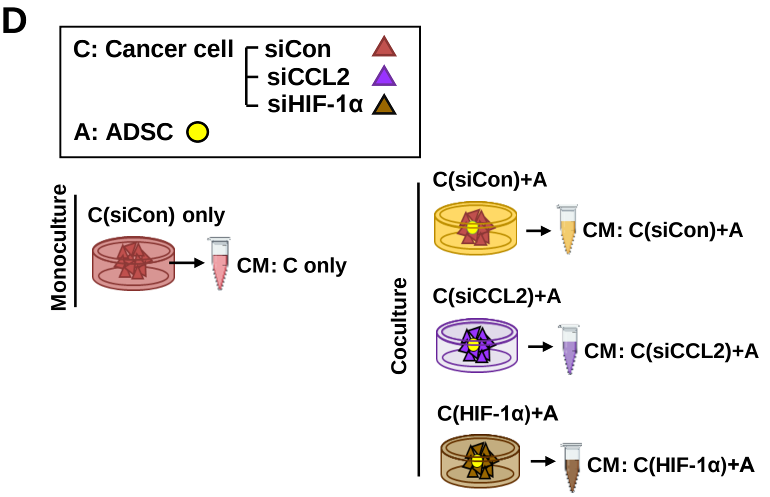

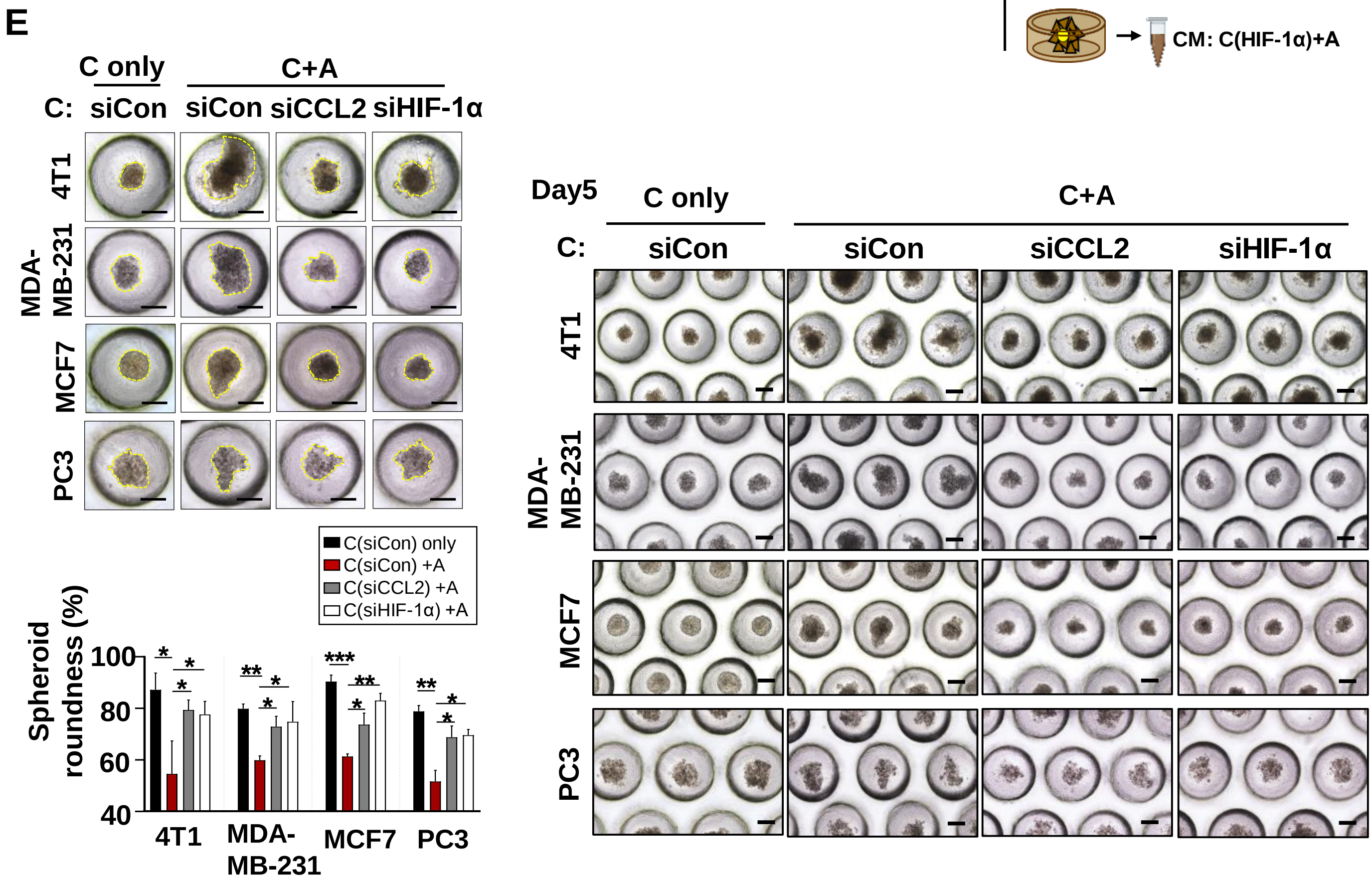

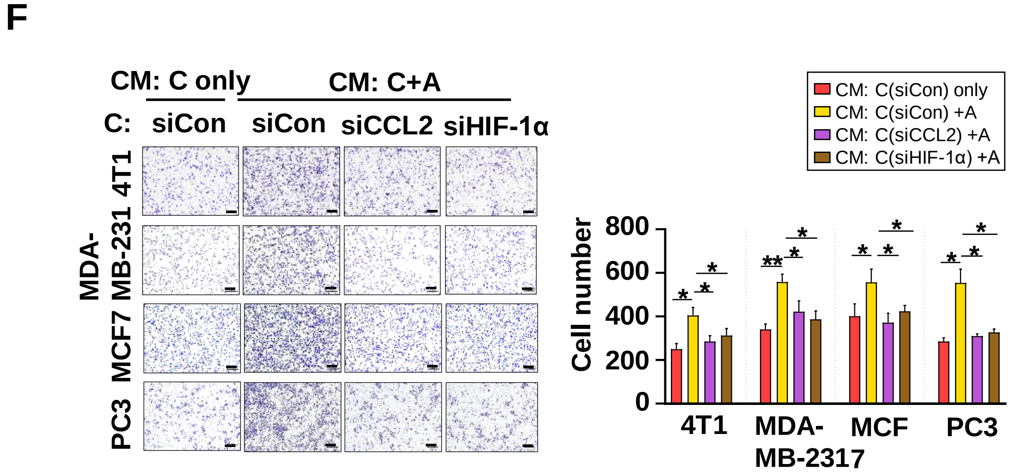

Supplementary Fig. 3 (Continue)

G

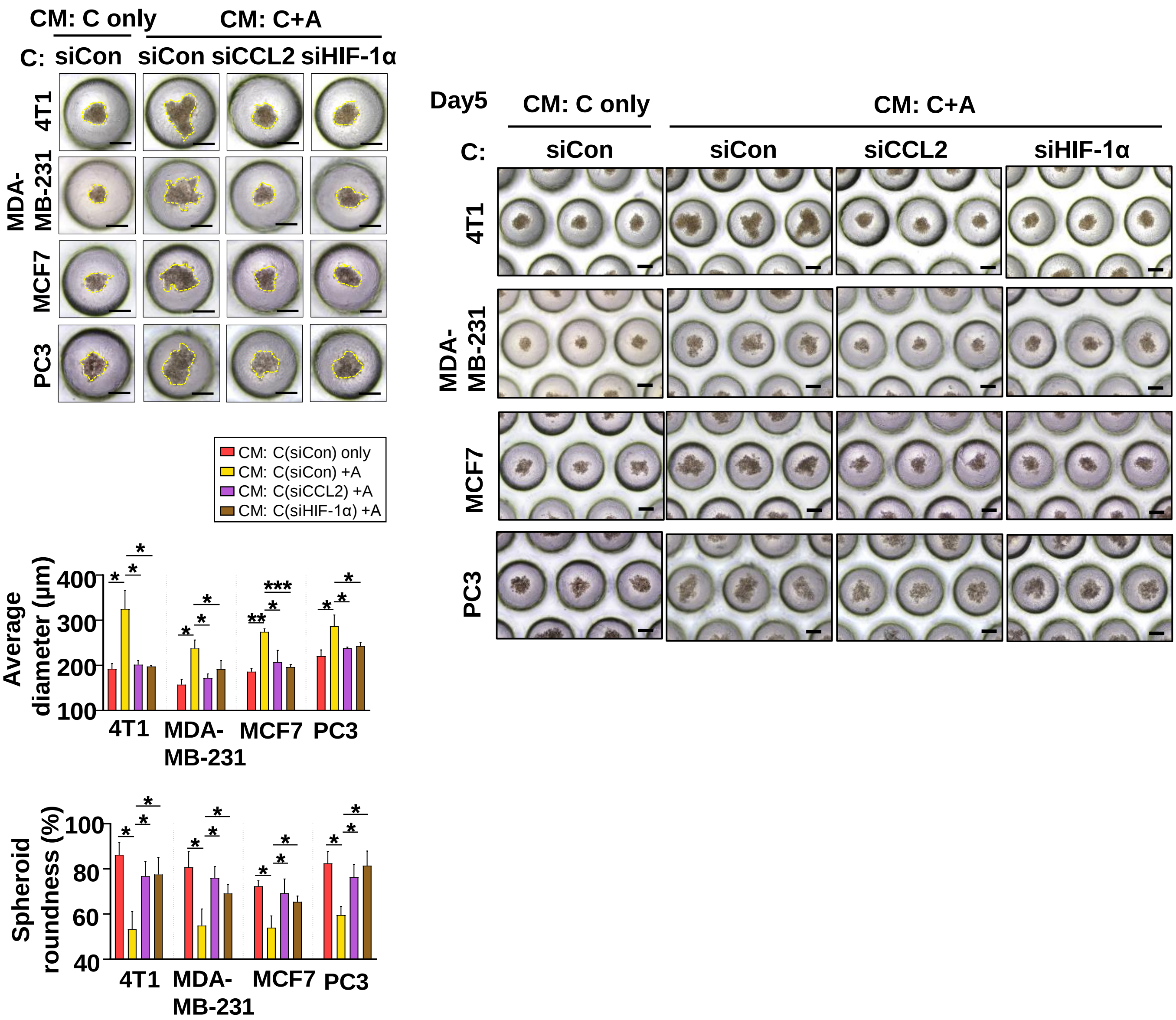

Supplementary Fig. 3 (Continue)

H

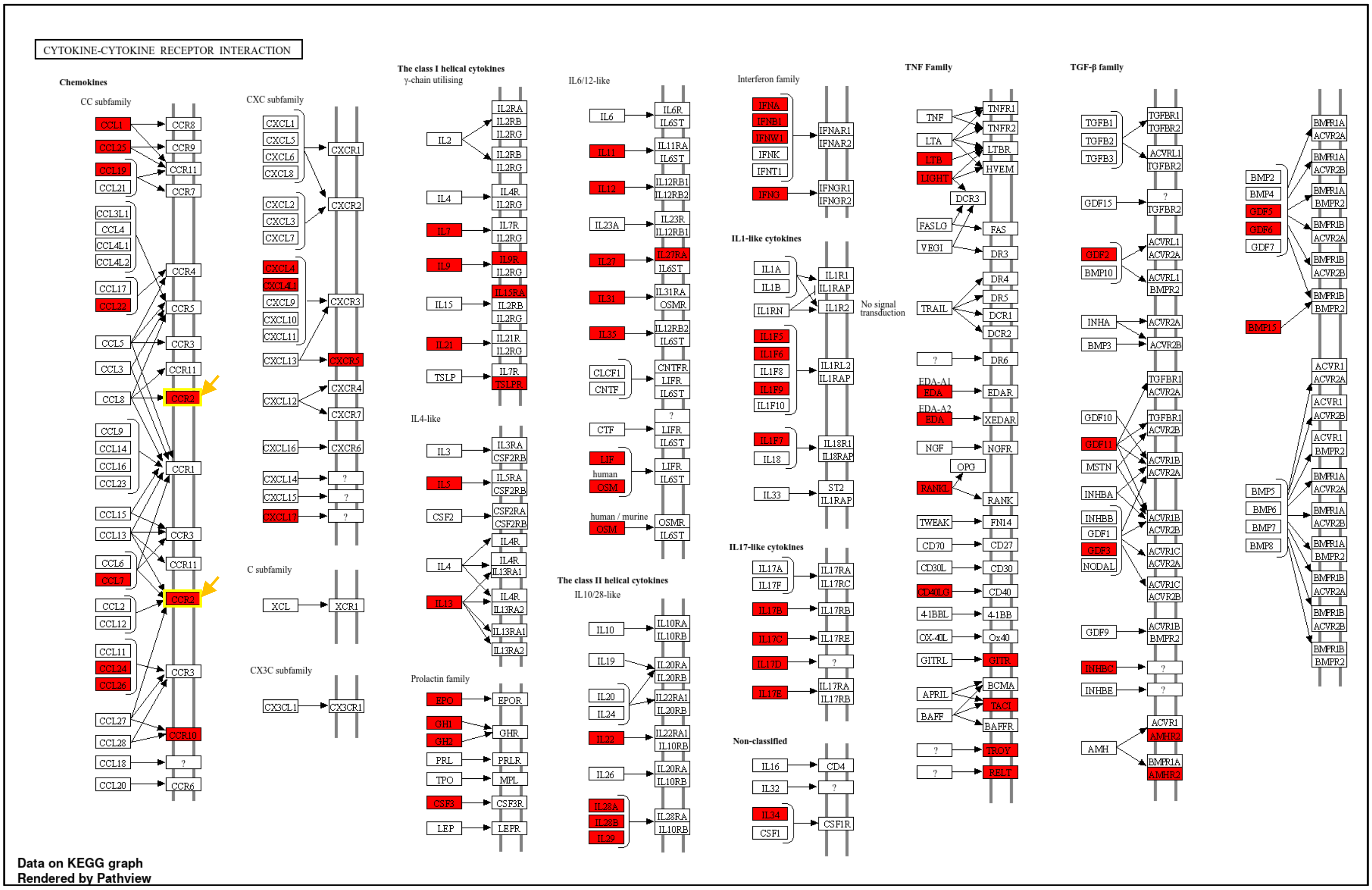

Supplementary Fig. 3 (Continue)

I

GSE153316
(Genes in Adipose Tissue of Obese Breast Cancer Patients)

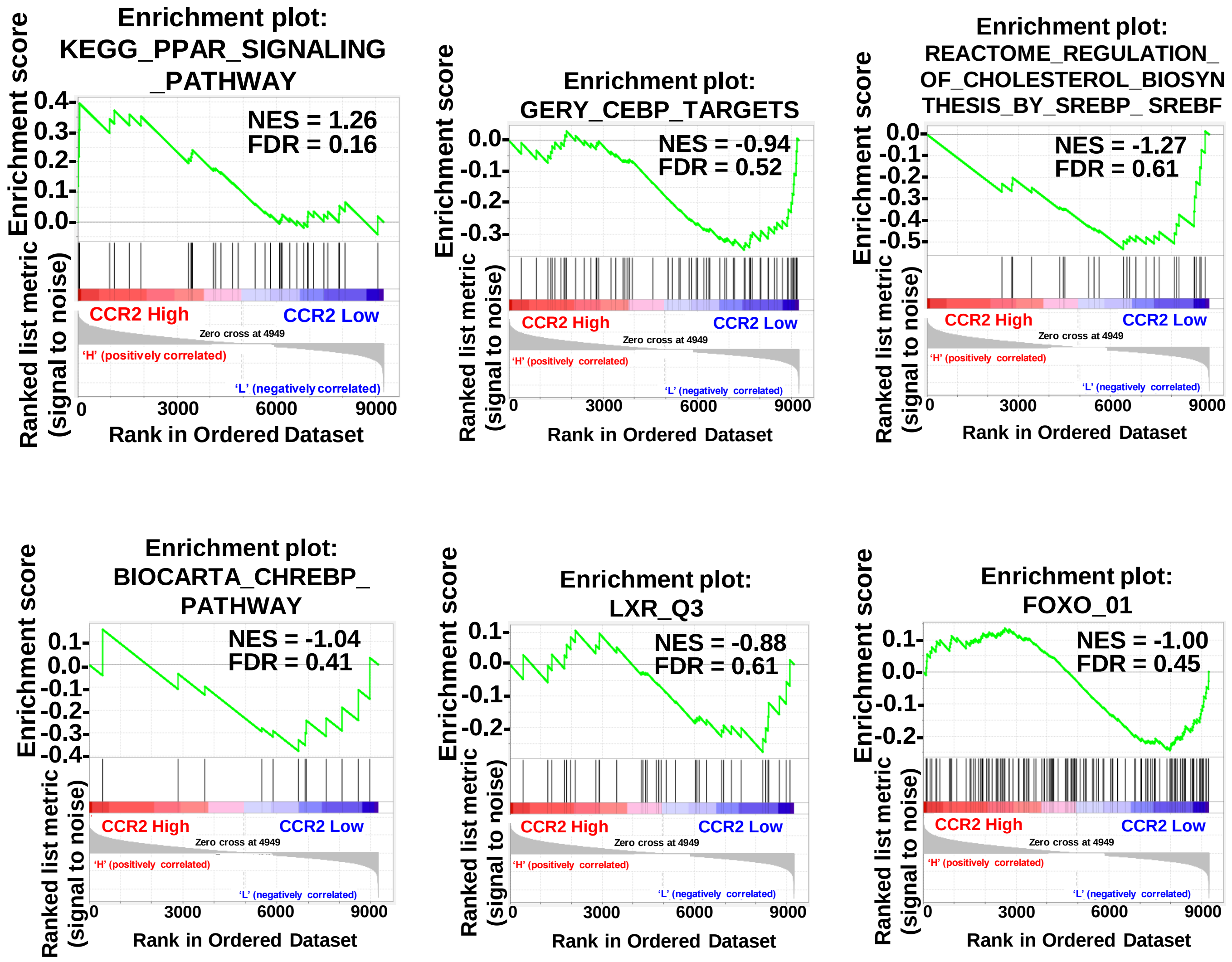

Supplementary Fig. 3 (Continue)

J

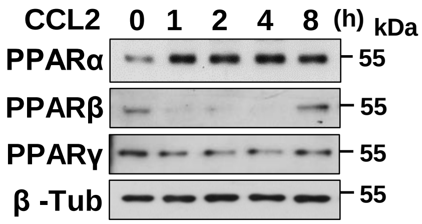

K

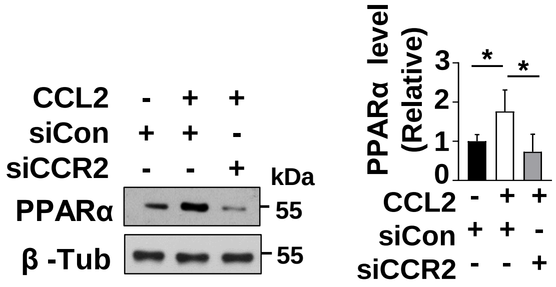

L

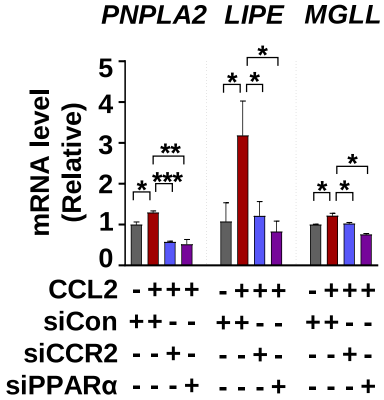

M

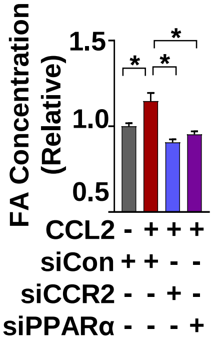

Supplementary Fig. 3 (Continue)

Supplementary Figure 3. Extended data for Fig.3 (Continue)
(A) Single-size (left) and Full-size (right) images of spheroids on 3D culture chips on the 5th day. The spheroid roundness was calculated as described. Scale bar = 200µm. Mean ± SD (n = 3); *, P < 0.05; **, P < 0.001; ***, P < 0.0001. The cell culture media were replaced with fresh media containing PBS or nAb-CCL2 every 48 hours, and the CM from each chip were collected every 48 hours and filtered through 0.45µm filters before being used for measurement of fatty acid (FA) concentration (Fig.3A) and transwell (Supplementary Fig.3B). Mouse neutralising antibody-CCL2 (nAb-CCL2) was treated to 4T1 cells (20µg/ml) and human nAb-CCL2 was treated to MDA-MB-231, MCF7, and PC3 cells (1µg/ml). (B) A transwell assay was performed by placing CM collected from the experiment shown in Supplementary Fig. 3A into the lower chamber. After 48 hours, migrated cells were quantified by counting the number of cells. Scale bar = 200µm. Mean ± SD (n = 3); *, P < 0.05; **, P < 0.001; ***, P < 0.0001. (C) Cancer cells were transfected with either siCon or siCCL2, and the expression level of CCL2 was measured by Western blot using indicated CCL2 antibodies to confirm whether the siRNA functions properly. (D) Schematic diagram for Supplementary Fig. 3E of mono- or co-culture 3D system with transfected cancer cells. (E) Single-size (left) and Full-size (right) images of spheroids on 3D culture chips on the 5th day. Cancer cells were transfected with siCon, siCCL2, or siHIF-1α and monocultured or cocultured with ADSCs at a 10:1 ratio. The spheroid roundness was calculated as described. Scale bar = 200µm. Mean ± SD (n = 3); *, P < 0.05; **, P < 0.001; ***, P < 0.0001. The cell culture media were replaced with fresh media every 48 hours, and the CM from each chip were collected every 48 hours and filtered through 0.45µm filters before being used for measurement of FA concentration (Fig.3B), transwell (Supplementary Fig.3F), and 3D culture (Supplementary Fig.3G). (F) A transwell assay was performed by placing CM collected from the experiment shown in Supplementary Fig. 3E into the lower chamber. After 48 hours, migrated cells were quantified by counting the number of cells. Scale bar = 200µm. Mean ± SD (n = 3); *, P < 0.05; **, P < 0.001; ***, P < 0.0001. (G) Single-size (left) and Full-size (right) images of spheroids on 3D culture chips on the 5th day. The same number of cancer cells were seeded on 3D chips and the CM from mono- or coculture of Supplementary Fig. 3E were treated for 5 days. The treated CM were replaced every 48 hours with newly obtained CM from Supplementary Fig. 3E. The average diameter of the spheroids was measured, and the spheroid roundness was calculated as described. Scale bar = 200µm. Mean ± SD (n = 3); *, P < 0.05; **, P < 0.001; ***, P < 0.0001. (H) KEGG pathway showing cytokine-cytokine receptor interaction (PATHWAY: hsa04060). Upregulated genes involved in cytokine–cytokine receptor interaction pathway of Fig. 3C are highlighted in red. Among them, CCR2 is specifically indicated with yellow arrows. (I) Genes of the NCBI GEO dataset (GSE153316) derived from adipose tissues of breast cancer patients with severe obesity (≥ 25 kg/m², n = 26) were divided into CCR2-high and CCR2-low expression groups. Gene set enrichment analysis (GSEA) plots present the relationship between CCR2 expression and downstream pathways of representative lipid-metabolism–regulating transcription factors, including the PPAR, C/EBP, SREBP, LXR, ChREBP, and FOXO1. (J) Human ADSCs were treated with CCL2 (20 ng/mL) for the indicated durations, and the levels of PPAR family (PPARα, PPARβ and PPARγ) were assessed by western blotting. (K) ADSCs were transfected with either siCon or siCCR2, followed by treatment with CCL2 (20 ng/mL). The protein levels were analysed by western blotting with indicated antibodies. Mean ± SD (n = 3); *, P < 0.05. (L) ADSCs were transfected as indicated, followed by treatment with CCL2 (20 ng/mL) for 24 hours. The mRNA levels were quantified by qRT-PCR. Mean ± SD (n = 3); *, P < 0.05; **, P < 0.001; ***, P < 0.0001. (M) ADSCs were transfected as indicated, followed by treatment with CCL2 (20 ng/mL) for 24 hours. FA concentrations in the CM were measured using the indicated FA quantification kit. Mean ± SD (n = 3); *, P < 0.05.

Supplementary Fig. 4

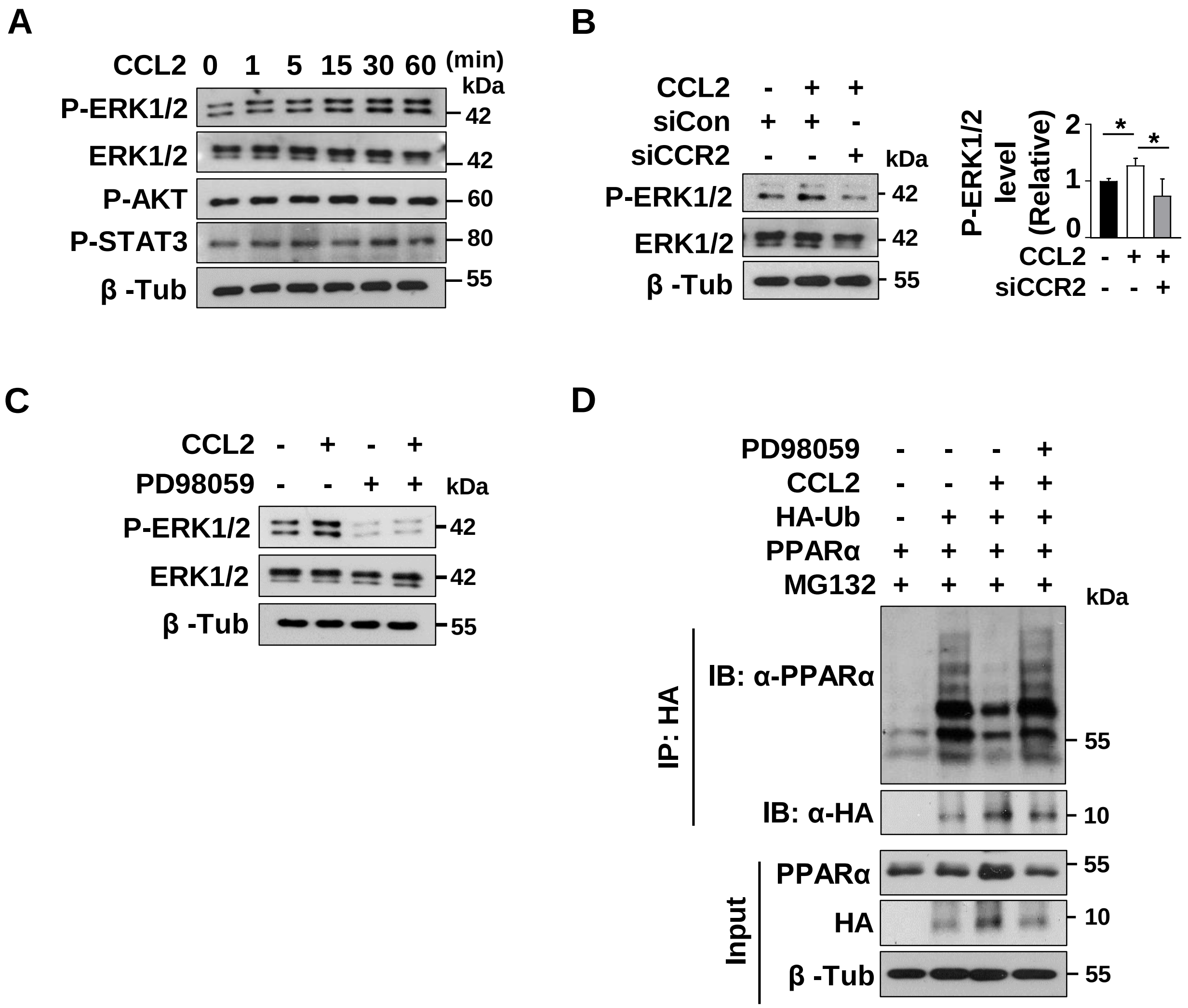

Supplementary Figure 4. Extended data for Fig.4

(A) Human ADSCs were treated with CCL2 (20 ng/mL) for indicated time points. Proteins were analysed by western blotting with indicated antibodies. (B) ADSCs were transfected with either siCon or siCCR2, followed by treatment with CCL2 (20 ng/mL) for 30min. Proteins were analysed by western blotting with indicated antibodies. Mean ± SD (n = 3); *, $P < 0.05$. (C) ADSCs were pre-treated with PD98059 (ERK1/2 inhibitor) (20 µM) for 4 hours and followed by treatment with CCL2 (20 ng/mL) for 30min. Proteins were analysed by western blotting with indicated antibodies. (D) ADSCs were transfected with HA-Ub and PPARα plasmid, followed by pre-treatment with PD98059 (20 µM) for 4 hours. Then, CCL2 (20 ng/mL) was treated for 4hours and MG132 (10µM) was treated for 2hours. Proteins were analysed by western blotting with indicated antibodies.

Supplementary Fig. 5

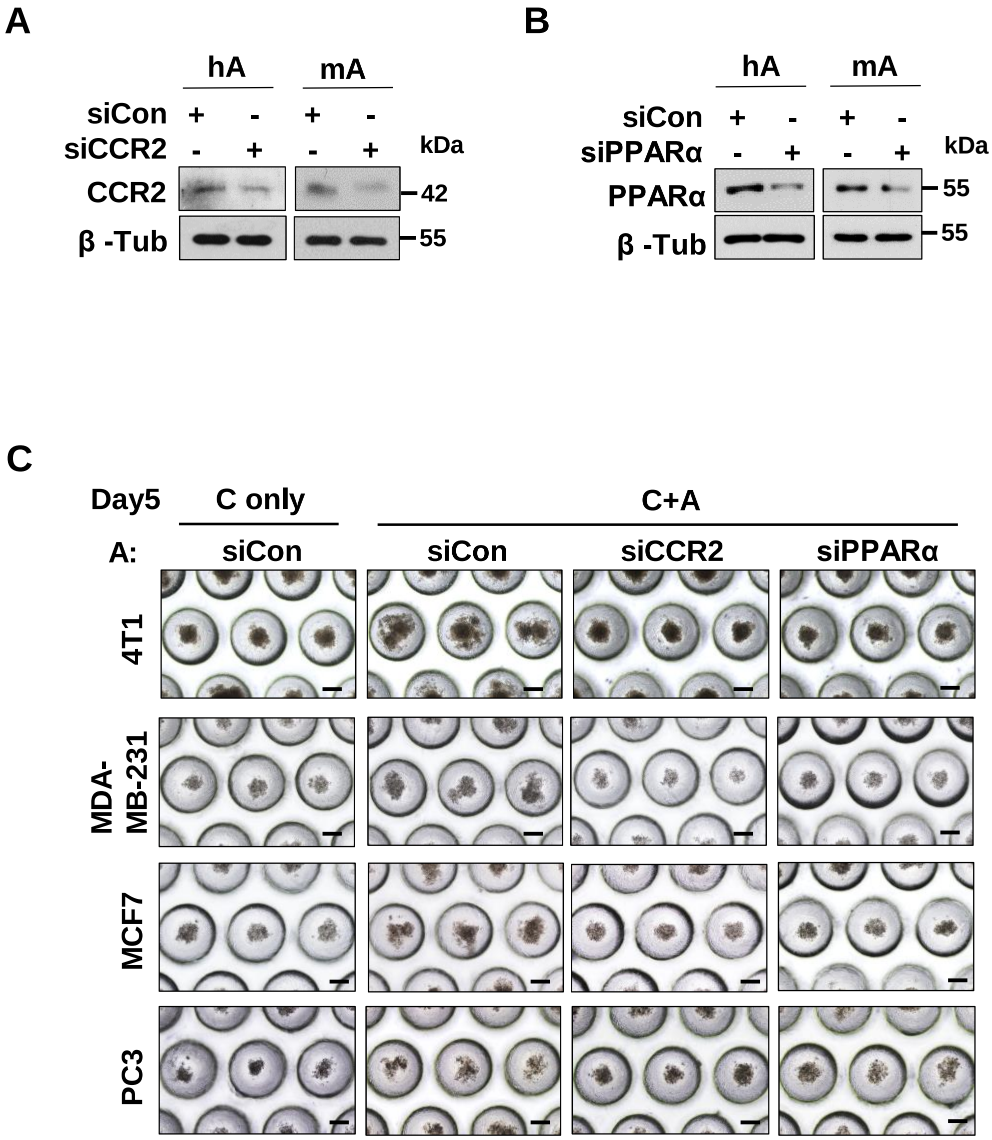

Supplementary Fig. 5 (Continue)

D

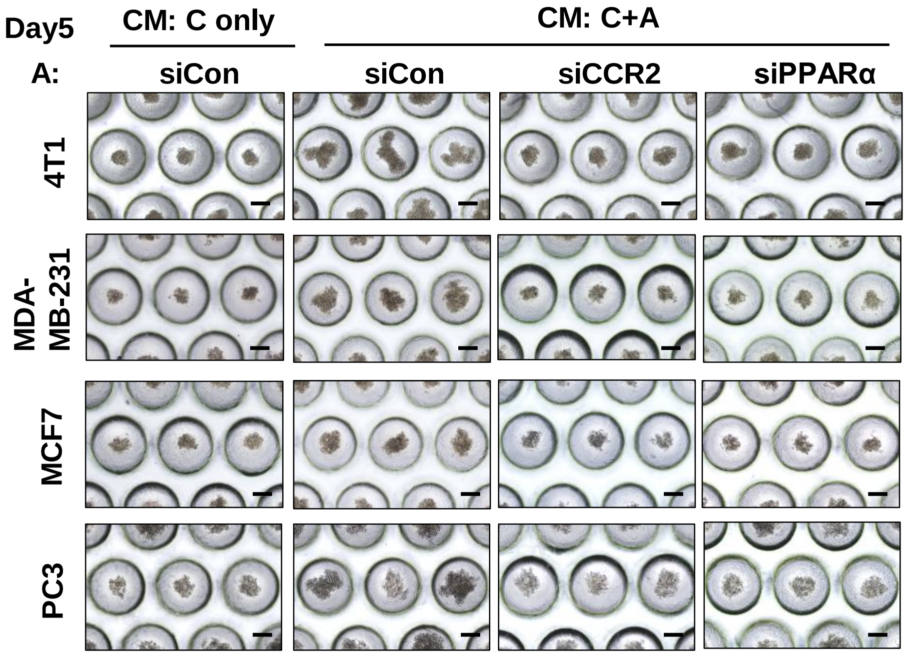

E

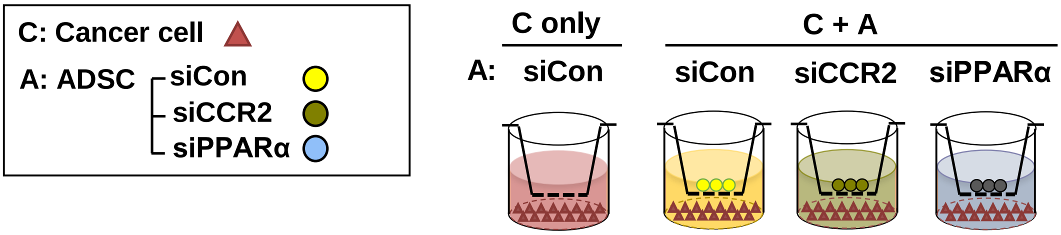

Supplementary Figure 5. Extended data for Fig.5

(A,B) Human ADSCs (hA) and mouse ADSCs (mA) were transfected with siCon, siCCR2, or siPPARα as indicated. Proteins were analysed using western blotting to confirm whether the siRNA functions properly. (C) Full-size images of cells on 3D culture chips on the 5th day (related to Fig.5B). Cancer cells were cocultured with ADSCs transfected with siCon, siCCR2, or siPPARα at a 10:1 ratio. The cell culture media were replaced with fresh media every 48 hours, and the collected CM from each chip were filtered through 0.45μm filters before being used for measurement of FA concentration of Fig.5C, transwell of Fig.5D, and 3D culture of Fig.5E . Scale bar = 200μm. (D) Full-size images of cells on 3D culture chips on the 5th day (related to Fig.5E). The treated CM were replaced with newly collected CM from the chips of supplementary Fig.5C every 48 hours. Scale bar = 200μm. (E) Structural and schematic illustration of indirect coculture. An indirect coculture system was established using a transwell insert with a 0.4 μm pore size, allowing the exchange of soluble factors through a shared medium while physically separating the two cell lines. Cancer cells were indirectly cocultured with ADSCs at a 5:1 ratio. Cancer cells were seeded in the bottom wells of a 12-well transwell plate, while ADSCs transfected with siCon, siCCR2, or siPPARα were cultured in the upper inserts to assess the coculture effects.

Supplementary Fig. 6

A

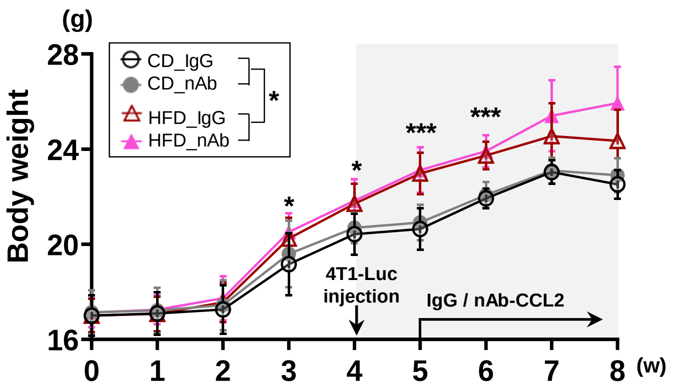

Supplementary Fig. 6

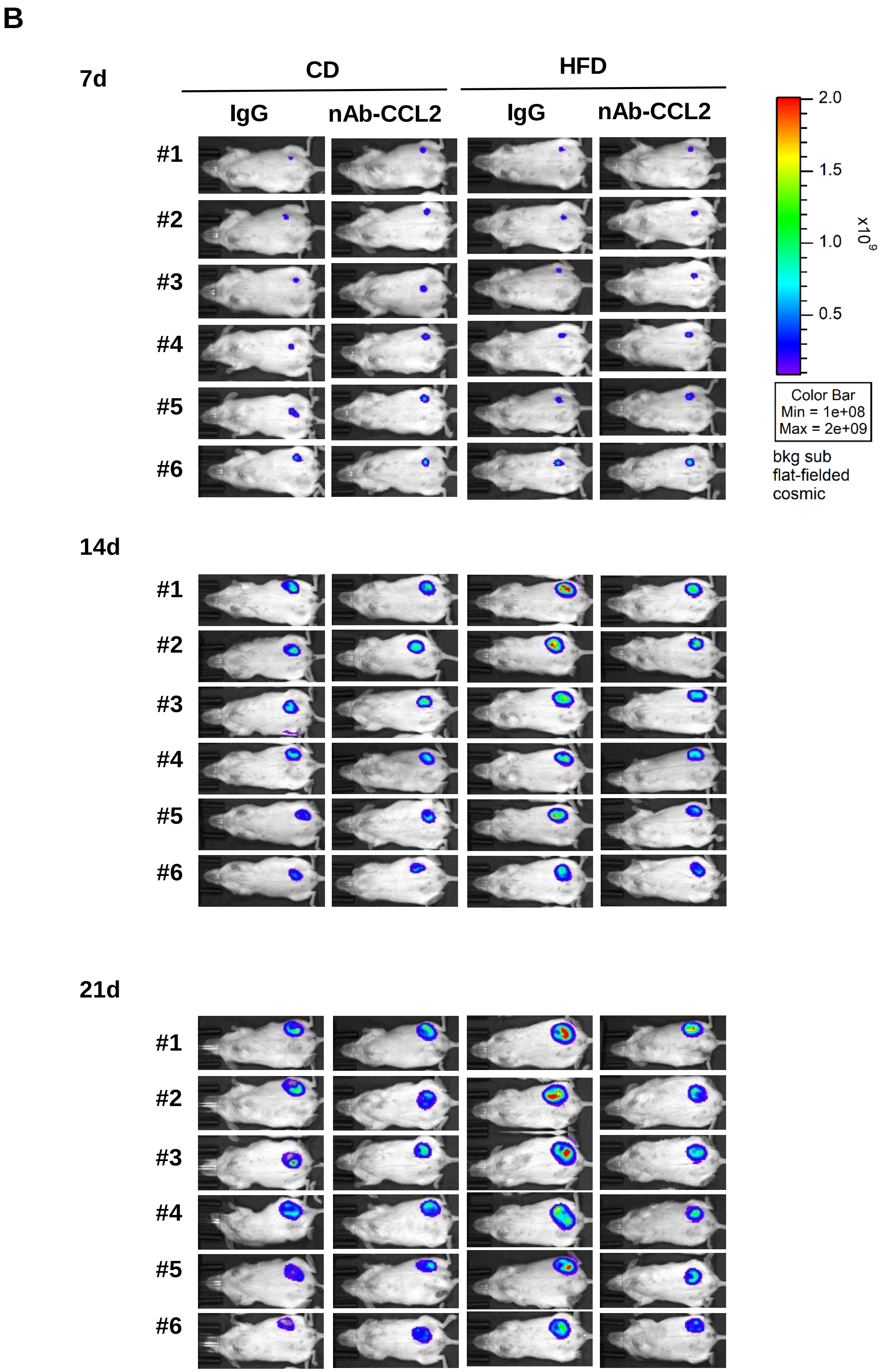

Supplementary Fig. 6 (Continue)

C

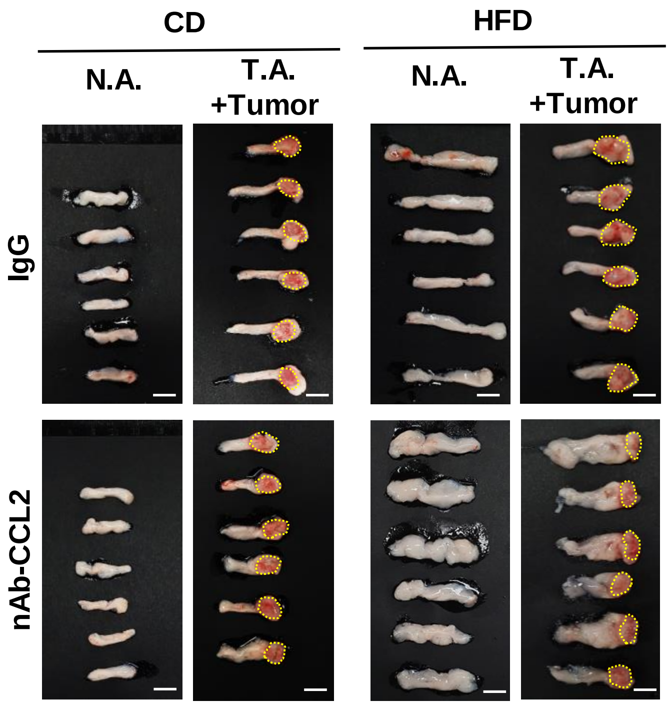

D

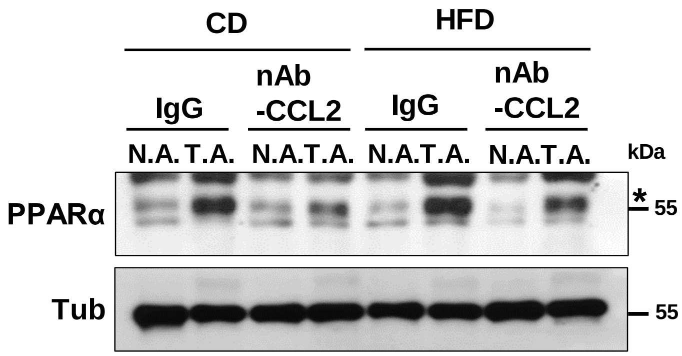

Supplementary Fig. 6 (Continue)

E

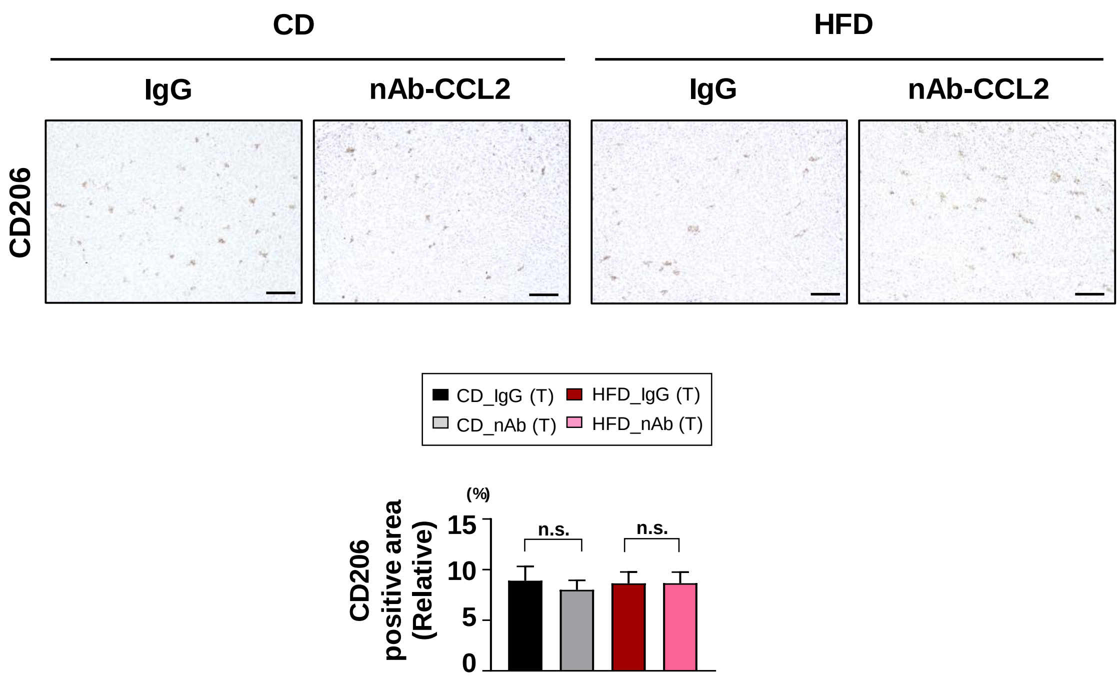

Supplementary Figure 6. Extended data for Fig.6

(A) Body weights were measured every week. Mean ± SD (n =6 in each group); *, P < 0.05; **, P < 0.001; ***, P < 0.0001; n.s., not significant (HFD group was compared with CD group). (B) Bioluminescence images of mice were obtained using the Xenogen IVIS® Lumina 100 every 7 days since cancer cells were injected, up to day 21. Colour scale bars represent luminescence intensity ranging from low (purple) to high (red) (n = 6 independent animals for each group). (C) The contralateral adipose tissue (non-tumor adipose tissue; N.A.) is a left fourth mammary fat pad of mice with no primary tumor, and the adjacent adipose tissue (tumor-adjacent adipose tissue; T.A.) is a right fourth mammary fat pad adjacent to primary tumor. Each tumor was collected along with the adjacent T.A. The outlines of each tumor were indicated by a yellow dashed line. Scale bar = 10 mm. (D) The PPARα protein expression levels of tumors collected from each group of mice were examined by western blot. (E) IHC of tumor sections using an antibody of M2 macrophage marker CD206 and DAB staining. Mean ± SD (n =6 in each group); n.s., not significant. Scale bar = 100µm.

Supplementary Tables

Supplementary Table 1. Oligo sequences for si-RNAs.

**Target gene**	**Sense (5' to 3')**
si-Control	AUGAACGUGAAUUGCUCAATT
si-HIF-1α (h)	GGGAUUAACUCAGUUUGAACUAACT
si-HIF-1α (m)	GAUAUGUUUACUAAAGGACAAGUCA
si-CCL2 (h)	CCCAAGAAUCUGCAGCUAACUUATT
si-CCL2 (m)	UGAAGCUAAUGCAUCCAUACCUTT
si-CCR2 (h)	GGAUUGAACAAGGACGCAUUUCCCC
si-CCR2 (m)	AACAUGUUGGUCAUUAUAAUUCUGA
si-PPARα (h)	GGGAAACAUCCAAGAGAUUUCGCAA
si-PPARα (m)	GAAAGUCCCUUAUCUGAAGAAUUCT
si-HUWE1 (h)	GAUUCUGAAGCAAGUAGUCAAUCAG

Supplementary Table 2. Primers used for RT-qPCR.

Target gene	Forward primer (5' to 3')	Target gene	Forward primer (5' to 3')
CCL7 (h)	F-ACAGAAGGACCACCAGTAGCCA<br>R-GGTGCTTCATAAAGTCCTGGACC	18S (h,m)	F-TTCGTATTGAGCCGCTAGA<br>R-CTTTCGCTCTGGTCCGTCTT
CCL2 (h)	F-GCTCAGCCAGATGCAATCA<br>R-TTTGCTTGTCCAGGTGGTC	Ccl2 (m)	F-GGGCCTGCTGTTCACAGTT<br>R-CCAGCCTACTCATTGGGAT
VEGFA (h)	F-CGTGTACGTTGGTGCCCGCT<br>R-CCGCTCTGAGCAAGGCCCAC	Vegfa (m)	F-GCTACTGCCGTCCGATTGA<br>R-CGCTTTCGTTTTTGACCCTT
PNPLA2 (h)	F-GATCACATCCTGGAGCACCT<br>R-ACAGGCAGCATGTTGGAGA	Pnpla2 (m)	F-TGCTGTGGTGGAGGAGAG<br>R-GTTGGAAAGGGTGGTCATC
LIPE (h)	F-GCTGCATAAGGGATGCTTCT<br>R-GAGATGGTCTGCAGGAATGG	Lipe (m)	F-ACTGAGATTGAGGTGCTGTC<br>R-AAGGCAGGTGAGATGGTAAC
MGLL (h)	F-GCTGGACCTGCTGGTGTT<br>R-CCTGACGAAAACGTGGAAGT	Mgll(m)	F-TGGCATGGTCCTGATTTCACCTCT<br>R-TTCAGCAGCTGTATGCCAAAGCAC
PCNA (h)	F-CATGGGCGTGAACCTCACC<br>R-CACAGCTGTACTCCTGTTCTGG	Pcna (m)	F-GATGCCGTCGGGTGAATTTG<br>R-TCTCTATGGTTACCGCCTCCT
CCNA1 (h)	F-CCTTAGGGAAATGGAGGTTAAA<br>R-CCAAATGCAGGGTCTCATTC	Ccna1 (m)	F-GCCTTCACCATTCATGTGGAT<br>R-TTGCTGCGGGTAAAGAGACAG
CCND1 (h)	F-TTCCTCTCCAAAATGCCAGA<br>R-CAGTCCGGGTCACACTTGAT	Ccnd1 (m)	F-GCGTACCCTGACACCAATCTC<br>R-CTCCTCTTCGCACTTCTGCTC
CCNE1 (h)	F-TCAGTGGTGCGACATAGAGAA<br>R-TGTCCAGCAAATCCAAGCTG	Ccne1 (m)	F-ACAAAGCCCAAGCAAAGAAA<br>R-GGCAGGTTTGGTCATTCTGT
BNIP3L (h)	F-TGCGAGGAAAATGAGCAGTC<br>R-TGCCATTGCTGCTGCTTTCATG	Bnip3l (m)	F-CCTCGTCTTCCATCCACAAT<br>R-GTCCCTGCTGGTATGCATCT
CCND2 (h)	F-GTGCTCCTCAATAGCCTG<br>R-TCTCTTTCGGCCCAACTG	Ccnd2 (m)	F-GAGTGGGAACTGGTAGTGTTG<br>R-CGCACAGAGCGATGAAGGT
PPARA (h)	F-CTATCATTTGCTGTGGAGATCG<br>R-AAGATATCGTCCGGGTGGTT	Ccng2 (m)	F-GGGGTTCAGCTTTTCGGATTG<br>R-AGATCAGCCCTTTTTCCCGAG
		Snai1 (m)	F-ATTCTCCTGCTCCCACTGC<br>R-GACTCTTGGTGCTTGTGGAG

Supplementary Table 3. The list of antibodies for immunoblotting.

**Antibodies**	**Source**	**Identifier**	**Bolcking buffer**	**Dilution**
Rabbit polyclonal anti-Ki-67	Novus Biologicals	NB500-170	1.5% BSA (IF)<br>3% BSA (IHC)	1:200 (IF)<br>1:500 (IHC)
Mouse monoclonal anti-CCL2	Invitrogen	MA5-17040	1.5% BSA (IF)<br>3% BSA (IHC, W.B.)	1:200 (IF)<br>1:250 (IHC)
Rabbit monoclonal anti-CCR2	Med Chem Express	HY-P80385	1% BSA + 1% skim milk (W.B.)<br>3% BSA (IHC)	1:500 (W.B.)<br>1:200 (IHC)
Rabbit polyclonal anti-PPARα	Gene Tex	GTX101098	1% BSA + 1% skim milk (W.B.)	1:1,000 (W.B.)
Mouse monoclonal anti-PPARα	Gene Tex	GTX12412	1% BSA + 1% skim milk (W.B.)	1:1,000 (W.B.)
Rabbit monoclonal anti-β-Tubulin	Cell Signaling	2146S	1% BSA + 1% skim milk (W.B.)	1:10,000 (W.B.)
Mouse monoclonal anti-Ubiquitin	Santa Cruz	sc-9133	1% BSA + 1% skim milk (W.B.)	1:1,000 (W.B.)
Rabbit polyclonal anti-P-ERK1/2	Cell Signaling	9101S	1% BSA + 1% skim milk (W.B.)	1:1,000 (W.B.)
Rabbit polyclonal anti-ERK	Cell Signaling	9102S	1% BSA + 1% skim milk (W.B.)	1:1,000 (W.B.)
Rabbit polyclonal anti-P-AKT	Cell Signaling	9271S	1% BSA + 1% skim milk (W.B.)	1:1,000 (W.B.)
Rabbit polyclonal anti-P-STAT3	Cell Signaling	9134S	1% BSA + 1% skim milk (W.B.)	1:1,000 (W.B.)
Rabbit polyclonal anti-HUWE1	Abcam	ab70161	1% BSA + 1% skim milk (W.B.)	1:1,000 (W.B.)
Rabbit polyclonal anti-HA	Santa Cruz	sc-805	5% skim milk (W.B.)	1:1,000 (W.B.)
Rabbit polyclonal anti-Phospho-PPARα (Ser12)	Invitrogen	PA1-820	5% skim milk (W.B.)	1:1,000 (W.B.)
Goat polyclonal anti-CD206	R&D Systems	AF2535	3% BSA (IHC)	1:200 (IHC)
Rabbit polyclonal anti-PPARβ	Santa Cruz	sc-7197	1% BSA + 1% skim milk (W.B.)	1:500 (W.B.)
Rabbit polyclonal anti-PPARγ	Santa Cruz	sc-7196	1% BSA + 1% skim milk (W.B.)	1:500 (W.B.)
Rabbit polyclonal anti-ATGL	ABclonal	A6245	3% BSA (IHC)	1:200 (IHC)

* W.B.: western blot

Supplementary Table 4. Clinical details of breast cancer patients used in analysis for IHC

No.	Age	Sex	Tissue	Diagnosis	BMI
**1**	63	Female	Breast Cancer	Normal	21.23742
**2**	39	Female	Breast Cancer	Normal	21.49811
**3**	53	Female	Breast Cancer	Overweight	23.04688
**4**	54	Female	Breast Cancer	Obese	25.39022
**5**	66	Female	Breast Cancer	Obese	26.68896
**6**	66	Female	Breast Cancer	Obese	26.68896
**7**	60	Female	Breast Cancer	Normal	19.37971
**8**	40	Female	Breast Cancer	Obese	26.19139
**9**	63	Female	Breast Cancer	Overweight	23.13616
**10**	79	Female	Breast Cancer	Obese	27.78695
**11**	63	Female	Breast Cancer	Obese	26.47837
**12**	49	Female	Breast Cancer	Normal	22.926
**13**	41	Female	Breast Cancer	Overweight	24.60938
**14**	39	Female	Breast Cancer	Overweight	24.82409
**15**	44	Female	Breast Cancer	Overweight	23.66129
**16**	34	Female	Breast Cancer	Normal	21.83597
**17**	61	Female	Breast Cancer	Normal	22.78616
**18**	57	Female	Breast Cancer	Normal	19.28081
**19**	47	Female	Breast Cancer	Obese	33.22127
**20**	42	Female	Breast Cancer	Obese	26.36674
**21**	52	Female	Breast Cancer	Normal	21.6435
**22**	37	Female	Breast Cancer	Normal	21.43659
**23**	52	Female	Breast Cancer	Normal	21.64769
**24**	43	Female	Breast Cancer	Normal	22.09197
**25**	32	Female	Breast Cancer	Normal	19.67355
**26**	50	Female	Breast Cancer	Obese	33.37784
